# Enhanced Refractive Index Sensitivity of Linearly Assembled Gold Nanoantennae for Biosensing Applications

**DOI:** 10.1002/smll.202510159

**Published:** 2025-12-23

**Authors:** Taufhik Hossain Tonmoy, Sezer Seçkin, Marisa Hoffmann, Isli Çela, Gyusang Yi, Ahmed Alsadig, Swagato Sarkar, Christian Rossner, Tobias A.F. König, Andreas Fery, Larysa Baraban

**Affiliations:** ^1^ Institute of Radiopharmaceutical Cancer Research Helmholtz‐Zentrum Dresden‐Rossendorf Dresden Germany; ^2^ Leibniz‐Institut für Polymerforschung Dresden e. V. Dresden Germany; ^3^ Else Kröner Fresenius Center For Digital Health Faculty of Medicine Carl Gustav Carus Technical University Dresden Dresden Germany; ^4^ Faculty of Chemistry and Food Chemistry Technische Universität Dresden Dresden Germany; ^5^ Department of Polymers University of Chemistry and Technology Prague Czech Republic; ^6^ Center for Advancing Electronics Dresden (Cfaed) Technische Universität Dresden Dresden Germany; ^7^ Dresden Center For Intelligent Materials (DCIM) Technische Universität Dresden Dresden Germany

**Keywords:** biosensors, gold nanoantennae, localized surface plasmon resonance, refractive index sensitivity, supracolloidal assemblies, Tumor Necrosis Factor Alpha (TNF‐α)

## Abstract

Compared to individual nanoparticles, supracolloidal structures offer unique plasmonic properties with enhanced susceptibility to variations in the local refractive index. In this study, we report the template‐assisted fabrication of large‐scale (*ca*. 1 cm^2^) linear periodic assemblies of AuNPs and demonstrate their polarization‐dependent biosensing performance at the proof‐of‐concept level. We explore two complementary approaches for template fabrication: i) laser interference lithography (LIL), offering high‐fidelity patterns over moderate areas, and ii) wrinkle‐assisted patterning, a more scalable and cost‐effective strategy. Despite structural differences, both approaches yield comparable sensing performances. This demonstrates the broad potential utility of the developed biosensing platform. First, sensors are characterized through varying concentrations of glycerol/water mixtures with known refractive indices. Next, as a proof‐of‐concept application, we perform biofunctionalization and detection of the antibody for Tumor Necrosis Factor‐Alpha (TNF‐α). TNF‐α is a pro‐inflammatory cytokine that plays a multifaceted role in cancer prognosis. The shifts in plasmonic resonance peaks of the AuNP assemblies are quantified using polarized‐light vis‐NIR spectrometry during various stages of the functionalization and detection process. The experimental results reveal a pronounced polarization‐dependent plasmonic resonance peak shift (Δλ). Overall, a signal enhancement of up to 5‐fold is observed in the longitudinal mode over the transversal case, a feature consistently achieved across both LIL and wrinkled templates. These findings present an innovative supracolloidal structuring approach for the development and scalable production of highly sensitive, plasmonic biochips.

## Graphical abstract

This study presents scalable methods for fabricating linear arrays of gold nanoparticles using template‐assisted self‐assembly. These supracolloidal structures show strong polarization‐dependent plasmonic responses for biosensing. Using antibody detection for Tumor Necrosis Factor‐Alpha (TNF‐α) as a proof‐of‐concept, the assemblies exhibit up to five‐fold plasmonic peak shifts in the longitudinally coupled case, highlighting their sensitivity and potential for developing advanced plasmonic biosensors.

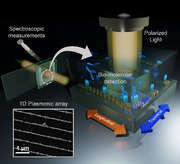

**Abbreviations**
AuNP: gold nanoparticle
BSA: bovine serum albumin
FDTD: finite‐difference time‐domain
GA: glutaraldehyde
LIL: laser interference lithography
LSPR: localized surface plasmon resonance
PDMS: polydimethylsiloxane
PEG: polyethylene glycol
SEM: scanning electron microscopy
TNF‐α: tumor necrosis factor‐alpha
vis‐NIR: visible to near‐infrared spectrometry

## 1 Introduction

Amongst the plethora of optical biosensing techniques reported to date [1, 2, 3, 4, 5, 6, 7, 8, 9], plasmonic sensors have garnered significant attention from the scientific community, owing to their exceptional sensitivity and potential as highly effective tools for rapid diagnostics in both research and clinical applications. Over the past few decades, surface plasmon resonance (SPR)‐based biosensors have become indispensable tools for probing biomolecular interactions in pharmaceutical and biomedical research [10]. These sensors are highly favored for their numerous advantages compared to many other label‐free optical techniques. These advantages include cost‐effectiveness, rapid sampling, label‐free detection, broad dynamic ranges, lower limit of detection (LOD), high sensitivity, and selectivity. SPR‐based sensors operating in the visible/near‐infrared wavelength range are of particular interest due to their favorable optical confinement at these wavelengths, positioning them as key components in the next generation of biosensing technologies [11]. However, despite serving as the reference standard for optically addressed sensors, conventional SPR biosensors are limited by the relatively long decay length of the evanescent fields (∼200–400 nm). This causes the sensor to average refractive index (n) changes over a relatively large volume, reducing its effectiveness in detecting low‐abundance biomolecules in small‐volume samples or near‐surface events. In contrast, localized surface plasmon resonance (LSPR), supported by plasmonic nanoantennae, offers substantial advantages [12] due to the confined evanescent near‐field around the particle. These fields decay over short distances, typically 5–30 nm, allowing for more precise detection of refractive index changes in the immediate vicinity of the nanoparticle surface. Additionally, LSPR spectroscopy offers wavelength tunability, reduced sensing volumes, and lower instrumentation costs [13]. Thus, LSPR‐based platforms are ideal for detecting biomolecules at the nanometallic surfaces [14]. Notably, gold nanoantennae (AuNPs) have been widely employed in diagnostics due to their unique optical, physicochemical, and electronic properties, which make them ideal for capturing and interacting with target analytes [15, 16, 17, 18, 19, 20]. Interestingly, the shape of AuNP affects their refractive‐index sensitivity [18]. Generally, the refractive‐index sensitivity scales with the LSPR wavelength [19, 20]. For example, the aspect ratio and size of gold nanorods may be tuned to display LSPR peaks at longer wavelengths. Gold nanorods having LSPR peaks around 800 nm can reach bulk sensitivity up to 600 nm/RIU [21].

While LSPR biosensors have demonstrated versatility, high detection sensitivity in the picomolar range, and the capability of detecting low‐molecular‐weight analytes [22], further advancements are required to fully realize their potential, particularly for ultrasensitive and specific applications, such as biomarker detection. Current limitations of LSPR‐based biosensing systems include small shifts (<10 nm) in resonant wavelength [23], broad spectral linewidth [24], low resonance intensity, and poor peak‐to‐valley contrast. All of these hinder the sensitivity and specificity of LSPR systems. Addressing these challenges necessitates the development of new strategies and nanoarchitectures to enhance plasmonic performance. In this regard, exploring the self‐assembly of varied geometric patterns [24, 25], nanoparticle clusters [26], and ordered arrays of plasmonic metal nanostructures or nanoparticles [27, 28, 29, 30] has emerged as a promising solution. These architectures can give rise to additional resonant modes that are highly tunable and exhibit enhanced field confinement, resulting in stronger and narrower resonance signatures than those of single nanoparticles. This has been demonstrated for core‐satellite type arrangement structures comprising 60 nm gold nanoparticle cores surrounded by 20 nm gold nanoparticle satellites. The arrangement structure resulted in refractive‐index sensitivity of 188 nm/RIU, higher than that of 60 nm gold nanospheres (112 nm/RIU) and 80 nm gold nanospheres (132 nm/RIU) [31]. In contrast to isolated particles, ordered arrangements of AuNPs exhibit plasmonic coupling, which is extremely sensitive to the interparticle distance [32]. The resulting near‐field coupling significantly enhances the localized electric field within the interparticle spacing, leading to a redshift of the LSPR wavelength. Such a spectral shift is not only a direct manifestation of the coupling but also serves as an optical sensor readout [33]. Several approaches have been developed to align AuNPs into 2D arrays using self‐assembly processes. For instance, Lalander et al. [34] reported on using DNA molecules as programmable scaffolds to guide the assembly of AuNPs onto nanostructured surfaces. By combining nanopatterning techniques, such as electron beam lithography, with DNA hybridization, highly ordered and reproducible nanoparticle assemblies were achieved. Despite its precision, the DNA‐directed assembly process still faces many challenges. One major limitation arises from the physicochemical properties of DNA when attached to the AuNPs. Furthermore, the high cost of functionalized DNA oligonucleotides makes DNA‐based fabrication quite challenging. Hanske et al. [35] demonstrated a simple, lithography‐free, scalable, and efficient approach for template‐assisted self‐assembly of monodisperse, protein‐functionalized AuNPs in wrinkle templates. This method enables the fabrication of highly uniform plasmonic arrays, presenting significant potential for adaptation into advanced sensing platforms. Capitalizing on the plasmonic coupling achieved through this uniform array approach, Schlicke et al. [36] recently reported substantial performance improvements in near‐infrared (NIR) photodetectors and photoactivated gas sensors through the incorporation of plasmonic nanoparticles. Nevertheless, despite these promising advancements, the application of such template‐assisted self‐assembly strategies in the context of biosensing has not been fully realized.

**FIGURE 1:**
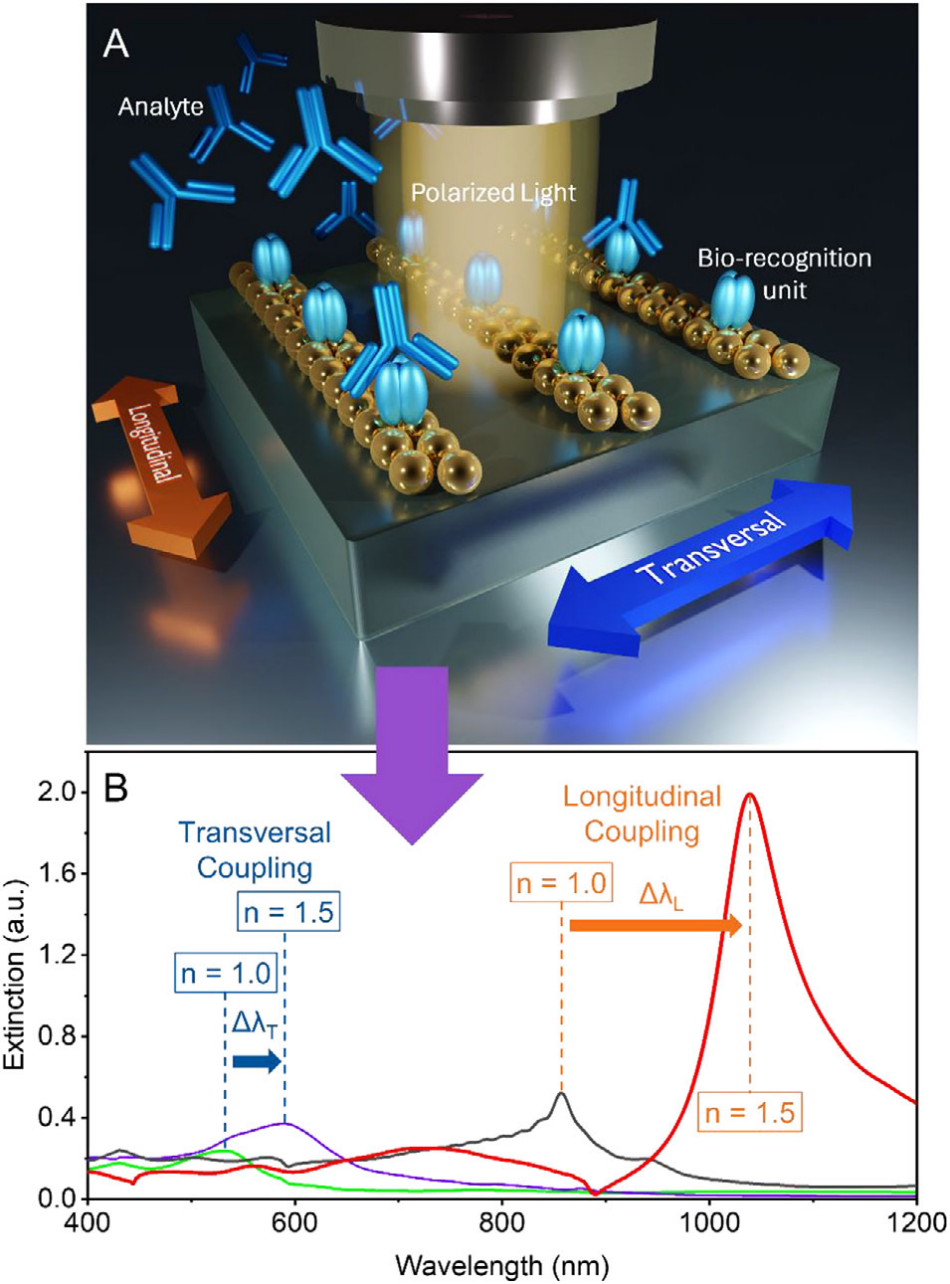
Schematic representation illustrating the proposed plasmonic biosensor using functionalized nanoparticle assembly. (A) Sensing concept, showing a bio‐recognition layer of Tumor Necrosis Factor‐alpha (TNF‐α) anchored to the surface of amine‐functionalized AuNPs within linear assemblies. A binding event involving the attachment of target biomolecules (anti‐TNF‐α) can change the local refractive index, n. (B) Simulations showing a redshift in the plasmonic resonance peaks due to changes in the refractive index. Δλ_T_ represents a shift in the resonance peak in the case of light polarized in the transversal direction, whereas Δλ_L_ corresponds to the longitudinal case.

Here, we fabricated periodic, linear assemblies of amine‐functionalized AuNPs on glass substrates via template‐assisted self‐assembly. Two distinct approaches were used for generating the PDMS templates: i) soft lithography based on laser interference lithography (LIL)‐defined masters and ii) oxygen‐plasma‐induced wrinkle formation on pre‐strained PDMS. These complementary patterning techniques both enable 1D structuring over large areas and were utilized to demonstrate the versatility and robustness of plasmonic nanoparticle chain formation. Initially, the prepared sensors were characterized through glycerol/water mixtures of varying concentrations. Furthermore, the quality factor of the resulting plasmonic resonances was characterized using the definition, Q‐factor = λ/FWHM, where λ is the resonant wavelength, and FWHM is the full width at half‐maximum of the resonance peak. Shifts in these resonance peaks were systematically monitored across different stages of bio‐functionalization using polarized vis‐NIR spectrometry, as illustrated in Figure 1. Both longitudinal (along the nanoparticle chains) and transversal (across the chains) plasmonic coupling modes were investigated. To further understand the plasmonic behavior and refractive index sensitivity, finite‐difference time‐domain (FDTD) simulations were performed for both transversal and longitudinal coupling directions. The increased refractive index sensitivity in the shift of the longitudinal coupling peak was experimentally validated by glycerol/water mixtures of known refractive indices. Furthermore, as a proof‐of‐concept, Tumor Necrosis Factor Alpha (TNF‐α) and its antibody (anti‐TNF‐α) were used. TNF‐α is a pro‐inflammatory cytokine with a multifaceted role in cancer research. It influences tumorigenesis, tumor progression, and responses to therapy. The antibody to this cytokine (anti‐TNF‐α) was used as a target analyte, demonstrating the potential of these engineered plasmonic arrays for sensitive biomarker detection in point‐of‐care (PoC) applications. Such sensors could facilitate response monitoring of anti‐TNF‐α therapies that are used against various autoimmune diseases, viral infections, and certain forms of cancer [37].

## 2 Results and Discussion

### 2.1 Fabrication of Template and Linear Periodic Assembly of AuNPs

**FIGURE 2:**
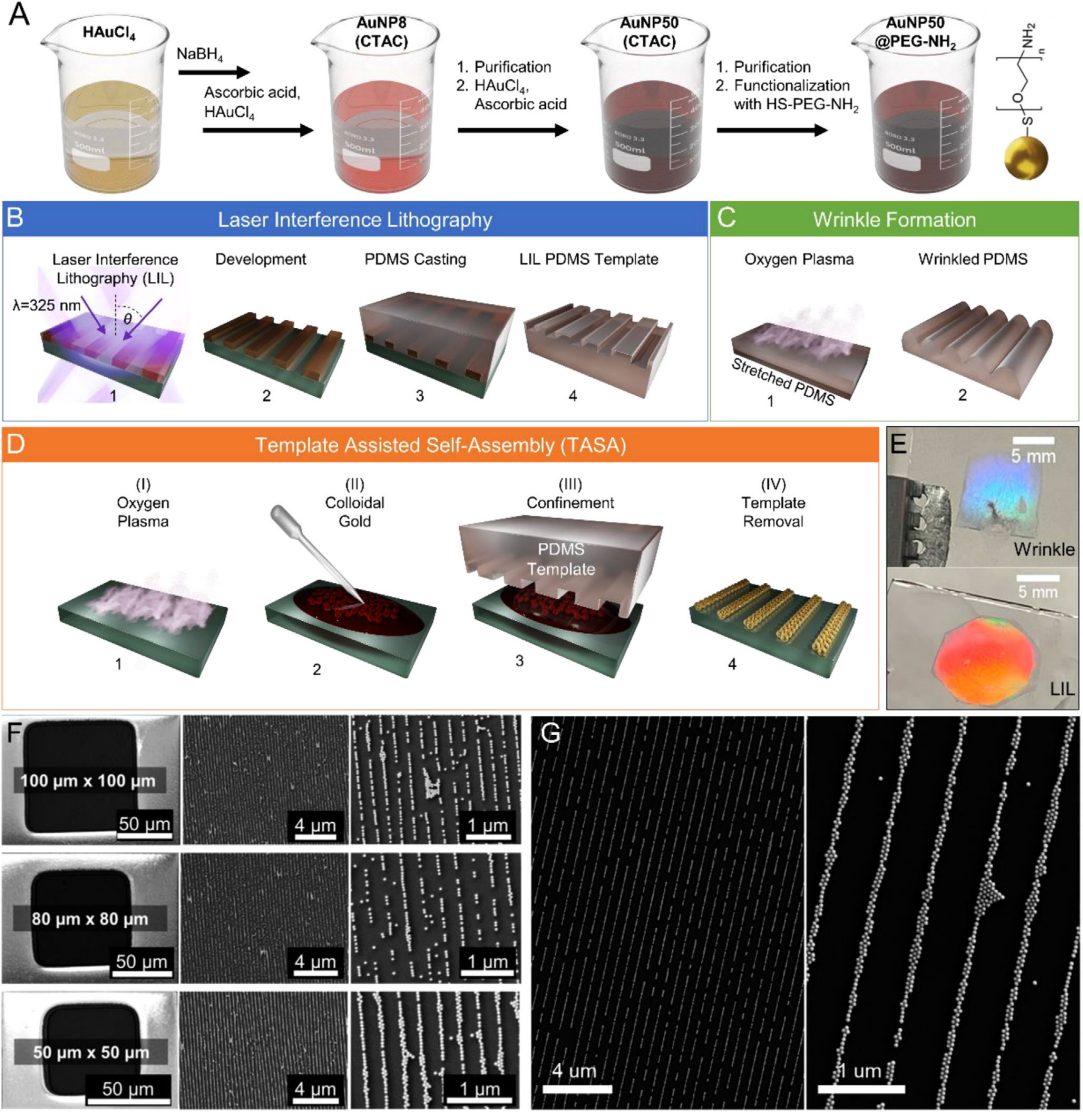
(A) Scheme for the synthesis and functionalization of dispersed gold nanoparticles (AuNPs). (B) Fabrication of templates using laser interference lithography (LIL). (C) Fabrication of wrinkled templates using oxygen plasma treatment of stretched polydimethylsiloxane (PDMS). Subsequent relaxation of the PDMS forms wrinkles due to a mismatch of Young's modulus between the bulk substrate and the plasma‐treated surface. (D) Illustration of template‐assisted self‐assembly (TASA) process. Plasma‐treated substrate is drop‐casted with colloidal AuNPs and dried under confinement of a PDMS template for self‐assembly into periodic arrays. (E) Photos of the resulting samples from two types of templates. (F) SEM images of single‐particle lines using wrinkled templates of 350 nm, and (G) dimer chains assembled with LIL templates of 590 nm periodicity. For SEM characterization, the assemblies were prepared on silicon wafers to facilitate imaging.

AuNPs synthesized using a modified version of a previously published protocol [38] were confined to linear chains using template‐assisted self‐assembly (TASA), as illustrated in Figure 2A–E. Details of the AuNP synthesis are provided in the Methods section. Microscopy glass slides were cleaned through ultrasonication in acetone, isopropyl alcohol (IPA), and water, respectively, for 10 min each and dried with a nitrogen gun. To enhance the surface hydrophilicity and promote uniform deposition of AuNPs, the substrates were treated with oxygen plasma (0.2 mbar, 80 W) for 10 min. 5 µl of concentrated amine‐functionalized AuNPs (2 mg ml^−1^) were placed on the substrate, covered with the polydimethylsiloxane (PDMS) template, and left to dry for 4 h. The template was removed, and the assembled particle lines on the glass were used for further experiments. Two different approaches were employed for fabricating the PDMS templates: i) laser interference lithography (LIL)‐based master structures, and ii) oxygen‐plasma‐induced wrinkle formation on pre‐strained PDMS.

The wrinkle‐assisted templates were fabricated using a 5:1 ratio of Sylgard 184 base to curing agent, following established protocols [39]. The oxygen plasma conditions used to obtain different wrinkle periodicities, along with a schematic of the fabrication process, are provided in the Supporting Information (Table S1 and Figure S2). Using 1 cm x 1 cm templates with 350 and 590 nm periodicities, AuNPs of ∼50 nm diameter were assembled into single and double‐particle (dimer) chains, respectively. Figure 2F illustrates such an assembly, highlighting that it is also possible to use the templates to form assemblies within small windows with topography (wells with 1.4 µm high photoresist walls in this figure). This method is scalable and offers a straightforward route for assembling nanoparticles over large areas using soft materials and readily accessible equipment, compared to traditional methods that often face scalability issues and require expensive equipment, such as electron beam or UV lithography for template fabrication. On the other hand, the LIL‐based templates were fabricated using a two‐beam interference lithography setup, as described in previous studies [40, 41]. Briefly, a laser beam was expanded, collimated, and split using a set of lenses and a beam splitter, respectively. These two beams were redirected using mirrors and made to interfere on a negative photoresist‐coated substrate. The exposed photoresist (exposure controlled through an electronic shutter) acquired a periodic pattern because of the constructive and destructive interference between these two beams. Intricate patterns can also be produced by increasing the number of beams, rotating the sample for multiple exposures [42], or using phase‐engineering techniques to manipulate the interference pattern [43]. The process involves exposing and developing the photoresist, followed by casting and curing PDMS, which together finalize the template fabrication. Further details are provided in the Supporting Information (Figure S3). This fabrication process enables precise pattern control and has been widely used to generate complex plasmonic and photonic architectures.[41] Figure 2G shows an assembly of dimer chains using a 590 nm periodicity LIL template. Both wrinkle‐assisted and LIL‐derived templates offer distinct advantages in terms of fabrication accessibility, structural fidelity, and area coverage. In this work, both techniques were employed to demonstrate the versatility of the template‐assisted assembly strategy for producing linear nanoparticle chains, which are suitable for optical and biosensing studies.

### 2.2 Refractive Index Sensitivity

**FIGURE 3:**
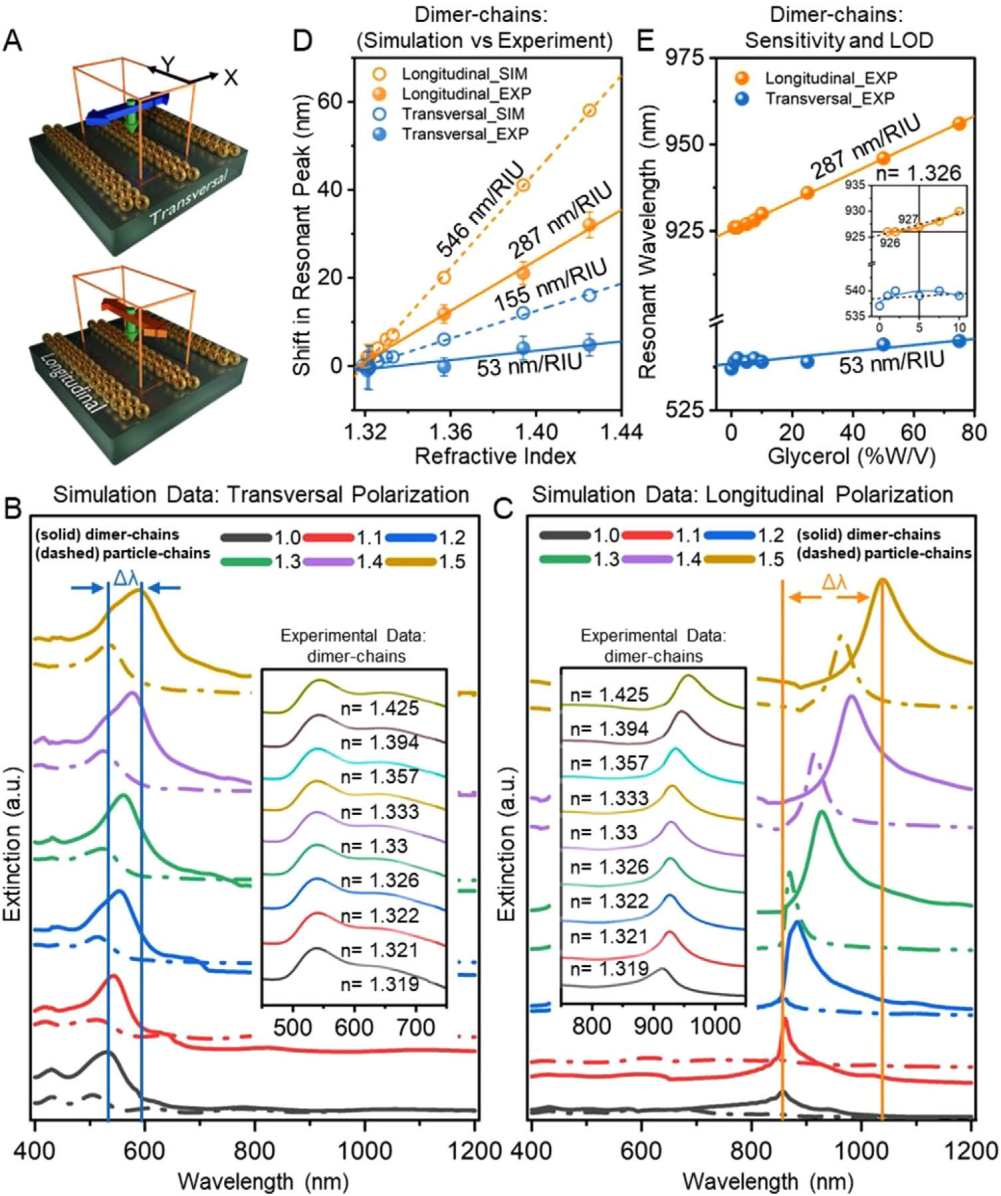
(A) Schematic showing x and y‐directions for the polarization of light for finite‐difference time‐domain (FDTD) simulations of nanoparticle arrays on a glass substrate. A chain length of 10 units is considered in each case. (B) FDTD simulation of the plasmonic response for 50 nm gold nanoparticles on a glass substrate when the refractive index (n) is changed from 1 to 1.5. Light is polarized in the transversal direction. Arrows represent changes in resonant peak position (Δλ). Dimer‐chains are represented with solid lines and single‐particle chains with dashed lines. The inset shows experimental data when various refractive indices are introduced through glycerol‐water mixtures in the case of a real sample of dimer‐chains assembly comprising polyethylene glycol‐coated gold nanoparticles with amine terminations (NH_2_‐PEG5k‐AuNP). (C) FDTD results for the same setup corresponding to light polarized in the longitudinal direction. The inset shows experimental data for the same sample in A but with light polarized in the longitudinal direction. (D) Comparison of the change in resonant peak position (Δλ) for the different light‐polarization cases. The suffixes _SIM and _EXP represent simulated and experimental results, respectively. (E) Resonant peak wavelength vs glycerol concentration (% w/v) graph showing the sensitivity and the limit of detection. For glycerol concentration changes smaller than 5%, corresponding to RI change (Δn) of 7*10‐3 RIU, the linear relationship was observed to break and the peak shift was insufficient (< 1 nm) for detection.

To understand the impact of refractive index variations on the LSPR modes of single *versus* assembled plasmonic particles, we conducted electromagnetic simulations using the FDTD method. Initially, we modeled a single spherical gold nanoparticle (AuNP) with a diameter of 50 nm placed on a glass substrate to assess its baseline optical response. This was tested in a background medium with a refractive index of 1 (air), which revealed an LSPR peak at approximately 512 nm. As we gradually increased the refractive index from 1 to 1.5, we observed a redshift of the dipolar LSPR mode from 512 to 552 nm (see Supporting Information, Figure S4A). To investigate how the number of plasmonic particles in the grating structure affects optical responses, we modeled an array of AuNPs arranged in dimer and single chains with a periodicity of 590 nm. Periodic boundary conditions were applied along the X and Y axes to mimic the periodic nature of the real structure (see Figure 3A). In contrast to the single‐particle case, we further extracted the optical response of these dimer‐ and single‐particle chains under two linear polarization orientations of the incident light: 90° (transversal coupling) and 0° (longitudinal coupling). The results are summarized in Figure 3B,C, respectively, displaying the plasmonic spectra shifts in the dimer (solid lines) and single (dashed lines) chains upon changing the refractive index of the surrounding media. In the case of transversal excitation, single‐particle chains and dimer‐chains exhibited resonances around 505 and 532 nm, respectively, when the refractive index of the surrounding medium was set to 1. Similar to the single‐particle resonance, these transversal modes of the particle chains experienced a red shift when the refractive index was changed from 1 to 1.5 (see Figure 3B). However, due to the close packing of the plasmonic particles in the dimer chains, the transverse mode incorporated both dipolar and quadrupolar coupling modes. This is evident as a shoulder in the extinction spectra [35, 44, 45].

Next, as the particle gratings were excited longitudinally along the chains, a broad and intense peak with a shoulder on the left side was observed for the near‐infrared region (Figure 3C). Since we modeled a quasi‐infinite number of particles along the chains, this intense peak referred to the hybridization of super‐ and sub‐radiant modes [35]. The resonant peak shift was found to be significantly greater in the case of longitudinal coupling compared to transversal coupling (182 nm vs. 57 nm for the dimer case and 138 nm vs. 28 nm for the single particle case). Furthermore, the longitudinal excitation of dimer particle chains led to stronger intensities in the extinction spectra of these modes, particularly when the refractive index was set between 1 and 1.3, as compared to single particle chains. Additionally, we observed that both transversal and longitudinal couplings in dimer‐chains resulted in larger redshifts compared to a single isolated particle (which showed polarization independence) and single‐particle chains (see Figure S4B). The overall resonant peak shifts during a change in refractive index from 1 to 1.5 for dimer chains were ca. 1.3 times and 2 times that of single‐particle chains for longitudinally and transversally excited cases, respectively (Figure 3B,C). The findings can be attributed to the increased plasmonic coupling effect and the phase retardation effect that occurs when the number of particles in the chain structures increases. This enhancement provides better sensitivity of these modes to changes in the refractive index [35, 44]. Therefore, in subsequent experimental studies, we focused on arrays of dimer‐chains.

For proof‐of‐concept, we used samples with dimer chains of 590 nm periodicity for molecular binding experiments. The feasibility of using AuNPs in biosensing necessitates the introduction of suitable terminal groups, such as amine or carboxylic, which can then be crosslinked to the desired capture probes. However, in practical scenarios, such surface modifications introduce an inherent heterogeneity in the local refractive index surrounding the AuNPs, even prior to the attachment of the desired biomolecules. Therefore, AuNPs covered by a shell of 5 kDa polyethylene glycol (PEG) with amine terminations were used to investigate their optical behavior in the vis‐NIR spectral range experimentally. First, the optical spectra and refractive index sensitivity of randomly drop‐casted particles were compared to those of particles assembled into dimer chains on a glass substrate. For spectral measurements, the setup (see Figure S5) consisted of the glass chip containing AuNPs and another clean glass slide with a thick double‐sided tape sandwiched between as a spacer to create a cavity for the placement of liquid. The cavity was then kept empty to represent air with a refractive index of 1.0 or filled with ∼250 µL of deuterated water (heavy water) at different concentrations of glycerol (for example, 0%, 25%, 50%, and 75% w/v) to tune the value of refractive indices [42]. The measured refractive indices of the mixtures, Cauchy distribution fits, and corresponding experimental data are provided in the Supporting Information (Table S6, Figure S7, and Table S8). For dimer‐chains having 590 nm periodicity, the transversal polarization showed only a slight red shift of ∼5 nm for a change in refractive index from 1.319 to 1.425 (Figure 3B, inset). In contrast, the longitudinal polarization of light excited strongly‐coupled peaks in the near‐infrared (NIR) region and showed a red shift of 32 nm for the same refractive index variation (Figure 3C, inset). As expected, the drop‐casted sample did not show dependence on the polarization of light, and the resonant peak in the visible range (544 nm) shifted by up to 11 nm upon refractive index variation (Figure S9). A linear fit of the experimental data provided a refractive index sensitivity of 100.56 nm/RIU for the drop‐cast sample. In contrast, dimer‐chains showed sensitivities of 53.15 nm/RIU and 286.97 nm/RIU for the transverse and longitudinal excitations, respectively. Hence, the experimental data were in good qualitative and quantitative agreement with the simulations, confirming that the dimer‐chains exhibit a significantly stronger refractive index sensitivity when excited longitudinally, compared to the polarization‐independent drop‐casted particles. Furthermore, the experimentally observed response under transversal excitation did not follow the simulation results. Our simulations predicted a stronger response from the transversal case of dimer‐chains than that of the polarization‐independent isolated particles (in our experimental case, the drop‐casted sample). We attribute this discrepancy to the possible swelling of the polymer ligands around the plasmonic particles during the refractive index sensitivity measurements, which affects the distance between adjacent plasmonic particles in both the transverse and longitudinal directions [46, 47]. As the interparticle distance increases, the transversely coupled dimer plasmonic modes undergo a blue shift [48], counteracting the red shift induced by the surrounding index change, resulting in a net shift that is much smaller than the predicted one. Furthermore, the drop‐casted sample showed a resonant wavelength above 600 nm in air, which suggests that some coupling effect was already taking place. However, it was polarization‐independent due to their random organization.

A noteworthy observation relevant to further biosensing data was made when the measurement environment was changed from a dry state (air) to water or glycerol mixtures. Despite the increase in refractive index environment from 1 (air) to 1.319 (deuterated water + 0% glycerol), a pronounced blue shift was observed instead of a red one in both regimes (see Figure S9). Several factors may contribute to this behavior. Among these is the refractive index correction due to the PEG‐NH_2_ hydrophilic linkers, which cover the AuNP particles (refractive index is 1.45 or higher in dry conditions vs. 1.33 in water), as well as their swelling when placed in an aqueous environment. Swelling of the PEG‐NH_2_ capping layer in an aqueous environment may modulate the plasmonic coupling between constituent nanoparticles [49], thus impacting the plasmonic spectra. This can even lead to a disruption of the plasmonic coupling between the dimers due to the potential fluctuating displacement of the particles along and across the lines [35, 46, 47, 50, 51]. To investigate further, FDTD simulations were carried out for dimer‐chains with increased interparticle distance in both x and y directions (Figure S10). It was observed that increasing the interparticle distance led to a blue‐shift in both transversal and longitudinal polarization cases. Therefore, the introduction of the fluid into the sample, *e.g*., in the first step of biofunctionalization, may impact the PEG linkers and the AuNP surface, as well as the integrity of the AuNP assembly, and thus the optical spectra measured in both transverse and longitudinal polarization modes.

In our glycerol‐deuterated water experiments, the linear slopes obtained through mean values of resonant peak shifts from multiple samples exhibited reliable R^2^ values and appropriate error bars (Figure S9D). These suggest that the assembled chips are suitable for reproducible results. Furthermore, repeatability within a single sample was also investigated. The dimer‐chains were exposed to multiple cycles of two distinct refractive indices (n = 1.357 and n = 1.425, corresponding to 25% and 75% glycerol (w/v) in deuterated water, respectively), and the results are presented in Supporting Information, Figure S11. The longitudinal case consistently showed a large shift in resonant peak, whereas the shift in the case of transversal polarization was observed to be very weak and could not distinguish such small changes in refractive index. (Figure S11A,B). The mean peak shift in the longitudinal case could be easily plotted to show a “switching” behavior (Figure S11C).

To summarize the characterization process, a comparison between simulation results vs experimental data was plotted for the dimer‐chains (Figure 3D), showing refractive index sensitivities of the longitudinal case to be 546 nm/RIU and 287 nm/RIU for simulated and experimental results, respectively. For the transversal case, the sensitivities were 155 nm/RIU and 53 nm/RIU for simulated and experimental results, respectively. To further fine‐tune the characterization process and showcase a performance of the plasmonic chip in biosensor mode, we extracted an “equivalent concentration resolution” parameter which may be analogous to the limit of detection (LOD) for the case of the glycerol/water mixtures. For this, the calibration curve was plotted for resonant peak vs glycerol concentrations for the dimer‐chains (Figure 3E). Considering the experimentally found refractive index sensitivity of 287 nm/RIU in case of longitudinal polarization, and 2.52 nm wavelength shift as uncertainty in individual measurements (observed from repeated measurements as shown in Supporting Information, Figure S11C), the smallest detectable glycerol concentration change can be estimated.

Considering refractive index resolution, δn = (2.52 nm)/(287 nm/RIU) = 8.78 ×10^−3^ RIU

Equivalent concentration resolution = δn/(Δn/ΔC)

$$=\frac{\left(8.78\times 10^{-3}\mathrm{RIU}\right)}{\left(1.425\mathrm{RIU}-1.319\mathrm{RIU}\right)\left(70-0\right)}$$

$$=5.8\%\;glycerol\left(w/v\right)$$

where, Δn = change in refractive index (RIU), obtained from Table S8

ΔC = change in glycerol concentration (% w/v)

Graphically, the curve in Figure 3E for the longitudinal case also exhibited a deviation from the linear relationship and indistinguishable peak‐shifts for glycerol concentration changes of approximately 5% (corresponding to a refractive index change, Δn, of 7×10^−3^). The value of 5.8% can therefore be considered a minimum detectable glycerol concentration change for practical purposes.

### 2.3 Biofunctionalization with Tumor Necrosis Factor‐Alpha (TNF‐α)

**FIGURE 4:**
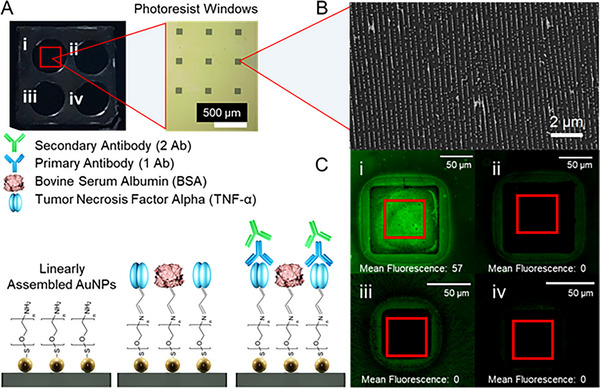
(A) Schematic representation of the functionalization protocol used for attachment of Tumor Necrosis Factor‐Alpha (TNF‐α). Gold nanoparticles with 5 kDa polyethylene glycol coating and amine‐termination (NH_2_‐PEG5K‐AuNP) were used for template‐assisted self‐assembly on a silicon substrate having small photoresist windows. Glutaraldehyde (GA) was used as a cross‐linker, while Bovine Serum Albumin (BSA) was used as a blocking agent to avoid non‐specific interactions. The functionalized surface was used to allow the primary antibody (anti‐TNF‐α) to bind specifically. Successful binding can be investigated through the attachment of a fluorescently labeled secondary antibody and subsequent fluorescence microscopy. (B) Scanning electron microscopy (SEM) images show nanoparticle chains within small photoresist windows on the silicon surface. (C) Fluorescence microscopy images (488 nm excitation, 5000 ms exposure) for the following conditions: (i) complete functionalization and detection sequence, GA, TNF‐α, BSA, primary antibody, secondary antibody; (ii) control sample with GA, BSA, primary antibody, secondary antibody (no TNF‐α); (iii) GA, TNF‐α immobilized with GA but without antibody treatment; (iv) original nanoparticle assembly without further processing.

To demonstrate the biosensing capabilities of our plasmonic platform, we used Tumor Necrosis Factor‐α (TNF‐α) and its binding to a primary antibody (anti‐TNF‐α) as a model. TNF‐α is a pro‐inflammatory cytokine whose expression is increased in various cancers, making it clinically relevant in the cancer framework. Owing to its well‐established association with tumor progression and therapeutic interventions, as well as its solubility characteristics, it is considered a promising candidate for exploration as a non‐invasive biomarker [52]. Immobilization of (TNF‐α) was achieved via an amine‐functionalized AuNP assembly placed on a silicon substrate. Glutaraldehyde (GA) was employed as a crosslinker between amine groups on the AuNP surface and those present on TNF‐α. Bovine serum albumin (BSA) was used as a blocking agent to prevent unspecific binding, while specific detection was achieved by binding a primary antibody (Anti‐TNF‐α). Initial confirmation of the successful binding was performed using fluorescence microscopy with a fluorescently labeled secondary antibody. After obtaining the amine‐functionalized AuNP assemblies on silicon wafers, the surface was compartmentalized using PDMS wells. A 5 mm diameter biopsy puncher was used to create 4 windows on a square‐shaped 15×15 mm PDMS section (Figure 4A). The PDMS wells were non‐covalently attached to the silicon chip with AuNP assembly (Figure 4B) by gently pressing them against the cleaned surface. Each well (W1 to W4) was assigned to a specific functionalization condition. W4 was kept untreated, and W2 was functionalized with BSA only, acting as a negative control for the functionalization. W3 was functionalized with TNF‐α but without primary antibody incubation, serving as a baseline for fluorescence microscopy using a 488 nm green filter. In W1, the full functionalization and detection steps were performed up to the incubation with the primary antibodies. To enable the detection of bound primary antibodies, the wells were incubated for 1 h in the dark with 5 µg ml^−1^ of a fluorescent‐tagged anti‐human IgG secondary antibody (SA5‐10110, Thermo Fisher). After washing, the wells were dried using a gentle air stream before being imaged for fluorescence. Further details of the functionalization process are provided in the Methods section. In case of successful functionalization and detection, testing with the secondary antibody should result in the accumulation of the fluorescent signal only in W1. As shown in Figure 4C, fluorescence emission was observed exclusively in W1, confirming successful functionalization with TNF‐α. The absence of fluorescence in W2 confirmed that the detection strategy was specific to TNF‐α, with no nonspecific binding of secondary antibody. As expected, W3 and W4 displayed no fluorescence signal, as these wells were not exposed to fluorescently labeled antibodies. Quantitative analysis and image processing were performed using ImageJ software, as detailed in Supporting Information (Figure S12).

### 2.4 Enhanced Biosensing Using Linear Assembly of Gold Nanoantennae

Exemplary biosensing experiments involving Tumor Necrosis Factor‐Alpha (TNF‐α) and its binding to primary antibodies were performed using AuNP assemblies on glass chips, using the same functionalization protocol as described in the Methods section.

In our design, the AuNPs were functionalized with SH‐PEG(5 kDa)‐NH_2_ prior to the template‐assisted self‐assembly. Despite PEGylation significantly minimizing biofouling through steric and hydration effects, the presence of terminal amine groups introduces a degree of surface reactivity that can still promote weak electrostatic or hydrogen‐bonding interactions in complex matrices, for example‐ with serum proteins in a biologically relevant media such as human serum. Therefore, to further minimize nonspecific adsorption, a blocking step using bovine serum albumin (BSA) was introduced after TNF‐α immobilization. BSA is generally used to block potential nonspecific adsorption sites. Recently, it has also been reported that BSA does not affect the binding constant between antibodies and thus serves its purpose well when only surface blocking is intended [53]. Furthermore, the use of bovine serum albumin (BSA) concentration of 1.5 mg mL^−1^ was also validated as sufficient for blocking the surface. In this case, an incubation of the dimer‐chains in BSA for 30 min resulted in a passivated surface that did not undergo any further significant resonant peak shift upon exposure to various concentrations of serum (see Figure S13).

To exclude any influence of varying biomolecule concentrations, we maintained a fixed concentration of the target analyte (anti‐TNF‐α) as well as the recognition unit (TNF‐α). The concentrations of TNF‐α (50 µg mL^−1^) and anti‐TNF‐α (5 µg mL^−1^) were chosen to remain clearly in the saturation plateau of the standard Enzyme‐Linked Immunosorbent Assay (ELISA) and ensure detectable interactions (see Figure S14).

The visible‐NIR spectra were obtained for linearly assembled dimer chains for three cases: (i) prior to functionalization, (ii) after immobilization of TNF‐α followed by surface passivation with bovine serum albumin (BSA), and (iii) after incubation with a primary antibody (anti‐TNF‐α) that selectively binds to TNF‐α. In addition to the enhanced refractive index sensitivity suggested by FDTD simulations, the dimer‐chains also offer better structural suitability than the single‐particle chains for practical experiments (see Figure 2F,G), due to a higher likelihood of continuous “filling” of the template, thereby avoiding breaks in the AuNP chains due to a single dislodged/missing particle.

**FIGURE 5:**
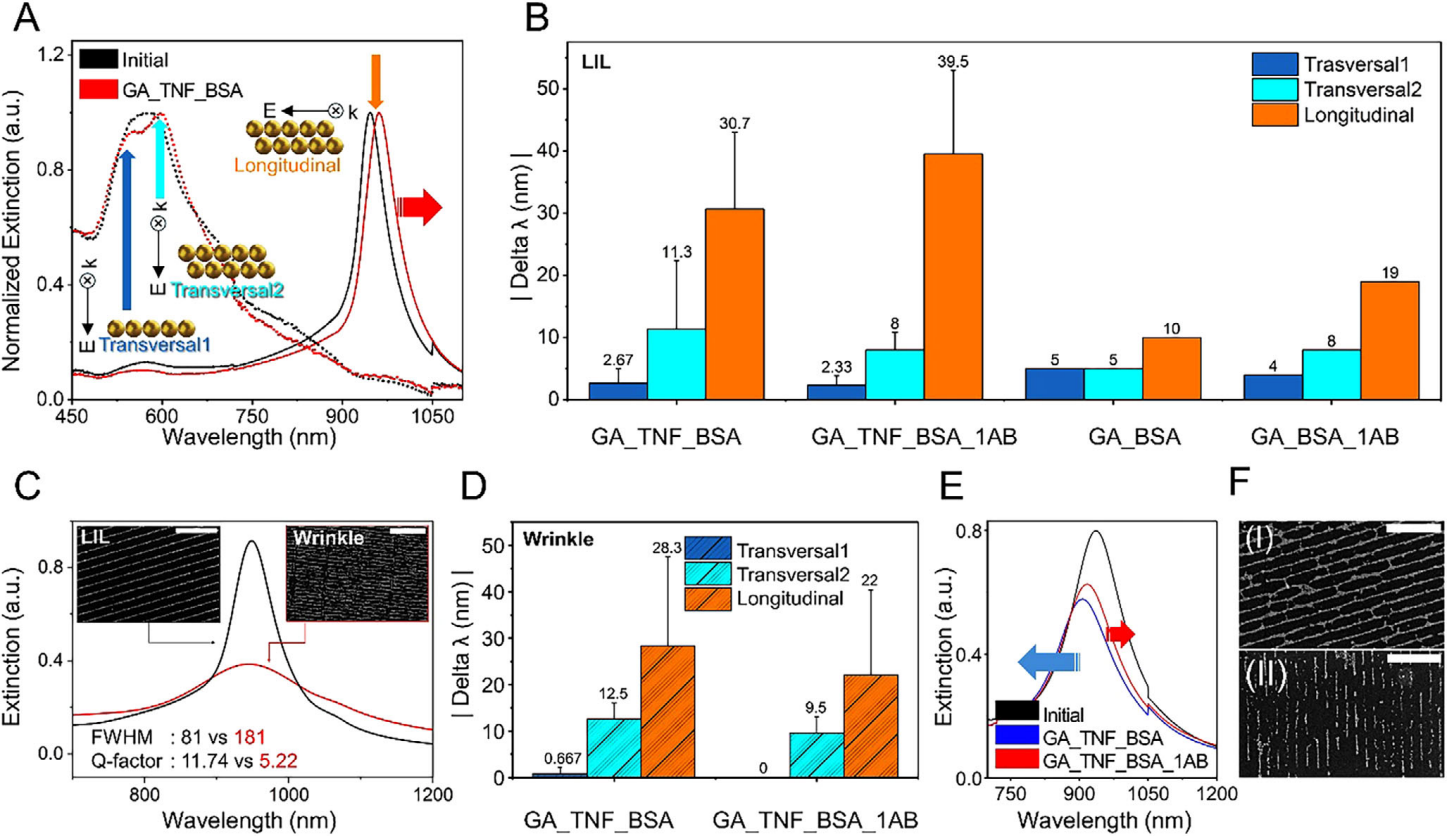
Vis‐NIR spectra of nanoparticle assembly and associated biosensing performance results. (A) Light polarized along the dimer chains shows a longitudinal peak, whereas light polarized across the chains shows two overlapping peaks‐ transversal1 and transversal2. The longitudinal peak exhibits a clear red shift from the initial case after the attachment of the biomolecule (Tumor necrosis factor, TNF‐α) and subsequent blocking with bovine serum albumin (BSA). (B) Change in resonant peaks for the various steps of functionalization, measured using a laser interference lithography (LIL) template‐assisted assembly of dimer‐chains with 590 nm periodicity. Δλ represents the absolute change in peak position from initial assembly to the respective steps labeled on the x‐axis. Notably, the transversal1 peaks were blue‐shifted and, therefore, absolute values were taken for simpler visualization. (C) Quality factor of assemblies prepared with a LIL template versus a wrinkled PDMS template. (D) Change in resonant peaks for the various steps of functionalization, measured using a wrinkled PDMS template‐assisted assembly of dimer chains. Same method of Δλ calculation. (E) Large blue shift after the GA_TNF_BSA step, followed by a small red shift after the primary antibody sensing step. (F) SEM images of the dimer‐chains showing removal of some particles after the experiment for the case of: (I) LIL template‐assisted assembly, (II) Wrinkled PDMS‐assisted assembly.

The biosensing capabilities and performance of the AuNP dimer‐chain patterns, fabricated by both wrinkled and LIL templates, were studied as illustrated in Figure 5. Measurements were carried out in air after each step to probe the effects of the functionalization and detection process. Otherwise, successive steps require different solvents and buffers, which could influence the background medium or affect the biomolecule layers.

In biosensing experiments with dimer‐chains assembled with LIL templates of 590 nm periodicity, a broad plasmonic peak was observed in the NIR region when the incident light was polarized in the longitudinal direction along the chains. For quasi‐infinite chains, as observed experimentally, a recent study has shown the formation of plasmonic bands as one moves from single to multiple particles, where the longitudinal mode is observed around 1.4 eV (∼900 nm) [28], for single particle chains. We observed such longitudinal modes around 1000 nm for the dimer chains, which also exhibited high‐quality extinction peaks due to lattice coupling. (Figure 5A). When the incident light was polarized in the transversal direction across the chains, the two overlapping peaks (transversal1 and transversal2) in the visible range were observed. The latter features potentially resulted from single particles and dipolar or quadrupolar coupling between the dimers [35, 51]. The resonant peaks shifted as subsequent steps of the functionalization process altered the refractive index environment near the particles, for both modes (red curves in Figure 5A). The experimental data (Figure 5B, LIL) agreed with the results from the simulations and refractive index sensitivity experiments presented in the previous section (Figure 3). It was observed that the biofunctionalization process (TNF‐α attachment followed by BSA blocking) caused significantly larger shifts (∼5‐fold) in the longitudinal peak in the near‐infrared region as compared to the resonant peaks from transversal polarization. The same trend was observed when the functionalized layer was used for sensing the analyte (i.e., binding of anti‐TNF‐α antibodies, labeled as 1AB). Therefore, exploring the longitudinal plasmonic modes in the linearly assembled AuNP supports an enhancement of the biosensing signal compared to the transversal modes when tested in identical conditions. A negative control, with no TNF‐α present in the functionalization step, was also measured. This time, the overall shifts in the longitudinal peak were smaller‐ 19 nm in the negative control vs 39.5 nm observed in the positive samples.

Notably, the large error bars indicate that there are significant sample‐to‐sample variations, and hence, the 19 nm shift in the case of the negative control is not a complete picture. The plots indicate absolute changes in the resonant peak position: |Delta λ| = |λ_step_ – λ_initial_|, where λ_initial_ and λ_step_ represent resonant peak positions of the original substrate with AuNP assemblies and the corresponding peaks after the functionalization/detection steps. Therefore, a larger number of steps (for example‐ GA_TNF_BSA_1AB) can potentially degrade the sample further than compared to lesser number of steps (GA_BSA_1AB). The degradation mechanism and resulting “blue‐shifts” are discussed further after presenting the results of the chips assembled with the second template‐ PDMS wrinkles.

Next to LIL, wrinkled PDMS templates offer a rapid and highly scalable method of assembling nanoparticles into chains [54, 55, 56, 57, 58]. Dimer chains assembled using this method also exhibited a strongly coupled plasmonic peak in the NIR region for longitudinal polarization. However, as illustrated in Figure 5C, the presence of cracks and inhomogeneities arising from the stretching/relaxation of the PDMS template required for wrinkles formation also resulted in defects in the dimer chains (Figure 5C, insets). These structural features may contribute to spectral variability and may limit reproducibility in biosensing scenarios. The full width at half maximum (FWHM) of the longitudinal resonant peak in the case of the wrinkled template (red curve in Figure 5C) was observed to be significantly broader (FWHM = 181 nm) compared to the peak of the chains assembled via the LIL template (FWHM = 81 nm). This resulted in peaks with different quality factors (Q‐factors) of 5.22 and 11.74 for the wrinkled and LIL templates, respectively, indicating that different fabrication conditions can influence the coupled plasmon resonances. This is particularly important in cases where small shifts in resonant peaks need to be detected precisely, since the higher Q‐factor supports much greater spectral resolution. Despite the relatively lower Q‐factor, the wrinkled PDMS‐assembled dimer‐chains were still effective in our proof‐of‐concept biosensing demonstration, showing the same trend that the resonant peaks shifted by a greater amount in the longitudinal cases than the transversal cases (Figure 5D). At the same time, a notable limitation of wrinkle‐based assemblies was the frequent occurrence of rather large blue‐shifts (≥10 nm) during biosensing experiments (Figure 5E, GA‐TNF‐BSA step), despite the red‐shift in the next functionalization step (Figure 5E, 1AB step), thereby complicating spectral interpretation. The undesired blue shifts were most frequently observed following the GA and BSA incubations.

As hypothesized previously in the refractive index sensitivity section, the initial blue shift after the GA step may result from the PEG hydration effect. Further blue shifts, prevalent for the BSA step, could be attributed to the removal of particles, which resulted in shorter AuNP chains and increased structural defects after these functionalization steps. Existing literature suggests that BSA may undergo conformational alterations affecting its secondary and tertiary structures upon binding to AuNPs. These changes result in a more flexible and less compact protein structure, exposing the hydrophobic cavities of BSA to the surrounding solution [59]. This could further explain why the BSA step is particularly prone to blue shifts, as conformational changes and further hydration could potentially facilitate the removal of particles from the chains.

To investigate the effects of particle removal, we performed further FDTD simulations. Keeping the periodic boundary conditions unchanged, particles were randomly removed from the dimer‐chains to generate plasmonic oligomers. Under these boundary conditions, a single unit cell containing 10 + 10 chains arranged in hexagonally close‐packed dimer lines was treated as being infinitely extended along both the x and y directions. Thus, below the finite chain limit of 10 particles [35], our simulations were performed using a total of 20 particles within one unit cell, representing an effectively infinite structure. Figure S15 presents FDTD simulation snapshots for different cases of particle removal, varying from 2 to 10 gold nanoparticles in increments of two, selected randomly from the unit. Simulations were performed for both air and 0% glycerol environments, as well as under transverse and longitudinal polarizations. As expected, particle removal caused negligible shifts in the transverse plasmon modes but resulted in a reduction in overall extinction intensity, primarily due to the decreased effective cross‐sectional area. In contrast, a distinct blue shift was observed, particularly in the 0% glycerol environment, indicating the emergence of plasmonic oligomer modes resulting from particle removal. For the air case, the extinction peak corresponding to these modes diminished and eventually vanished, confirming that the oligomeric plasmon modes are highly sensitive to the refractive index of the surrounding medium and persist only in higher‐index environments. Therefore, the hydration‐induced PEG‐swelling as well as further particle removal in successive steps could explain the rather small difference in resonant peak shift at the final samples (GA_TNF_BSA_1AB) vs the control samples (GA_BSA_1AB).

Overall, wrinkle‐assisted assemblies were observed to be more susceptible to particle removal during the biofunctionalization process. We speculate that this could be due to the inherent structural features, such as cracks perpendicular to the sinusoidal cavities of the wrinkles, as well as potential differences in particle filling compared to the LIL templates. SEM images support our interpretation, suggesting that the samples prepared with LIL templates remained more structurally intact after experimentation as compared to those prepared with wrinkled templates, although both suffered some degradation (see Figure 5C,F). These challenges, however, can potentially be addressed through improvement of the dimer‐chain stability on the substrate.

Whereas the dimer‐chain assembly in this work shows great potential for enhanced sensitivity utilizing the longitudinally coupled plasmon resonance peaks, various optimization possibilities are open for exploration to bring the proof‐of‐concept to a robust, practical sensing platform. These include: i) an additional drying step in a vacuum oven after the template‐assisted self‐assembly to improve adhesion of the particles to the substrate, ii) optimization of substrate cleaning protocols and plasma parameters before the particle assembly, iii) changing ligand chemistry on the nanoparticle surface for better adhesion to the substrate, iv) covalent attachment of the particles to the substrate and/or v) bringing the biorecognition element even closer to the nanoparticle surface, for example‐ using aptamers and DNA [60].

## 3 Conclusions

In this study, we provided critical insights into the strategic arrangement of spherical gold nanoparticles (AuNPs) to harness coupled plasmonic resonances for enhanced signal transduction, specifically in sensing applications. Template‐assisted self‐assembly was employed for the array formations, using two different approaches for template fabrication: i) laser interference lithography (LIL) and ii) wrinkled PDMS. Combined simulation and extensive experimental characterization using glycerol‐deuterated water mixtures of known refractive indices as well as biomolecular binding demonstrated that linear assemblies of AuNP dimers consistently exhibit enhanced refractive index sensitivity compared to the individual nanoparticles or drop‐casted layers. This enhancement stems from the plasmonic coupling under longitudinally polarized light, which leads to the hybridization of the plasmonic resonances to form super‐ and sub‐radiant modes, providing a compelling avenue for advancing refractive index‐based plasmonic sensors. Thus, assembling spherical AuNPs into supracolloidal structures on surfaces is a promising strategy for maximizing refractive index sensitivity that avoids the synthesis of special AuNP shapes.

For the biosensing proof‐of‐concept studies, specific interaction between Tumor Necrosis Factor‐alpha (TNF‐α) and its antibody (anti‐ TNF‐α) was used as a model. Furthermore, the bovine serum albumin (BSA)‐passivated sensors showed resilience to exposure with human serum, which suggests compatibility with complex physiological samples. Chips assembled through both the LIL and wrinkled PDMS templates were successful at achieving significantly stronger LSPR peak shifts (∼5‐fold) when excited with longitudinally polarized light as compared to transversal configurations. While LIL‐based assemblies showed narrower plasmonic resonances, resulting in higher Q‐factors, which support greater spectral resolution and precise peak discrimination, the template fabrication methods remain costly and time‐consuming. Alternatively, arrays formed by wrinkled‐PDMS template provide a rapid and scalable approach that does not require sophisticated lithography or metal evaporation/sputtering equipment, still offering the enhanced plasmonic shifts upon biomolecular binding. In addition, the tunability of resonance peaks, ranging from visible (∼530–545 nm for single particles in dispersion/transversal coupling in chains) to the near‐infrared (∼900–1000 nm for longitudinal modes of the dimer chains), enables optical selection for target‐specific excitation. These developments pave the way toward the next‐generation optical and optoelectronic devices and highly sensitive detectors. In particular, our findings open up an avenue for the development of ultrasensitive biochips in a cost‐efficient and scalable manner, leveraging a bottom‐up approach.

## 4 Experimental Section

### 4.1 Materials

Hexadecyltrimethylammonium chloride (CTAC, 25 wt.% in H_2_O), L‐( +)‐ascorbic acid (AA, > 99%), sodium borohydride (NaBH_4_, 99%), glutaraldehyde (GA, 25% in H_2_O), 2‐propanol (IPA,>99.5%), acetone (>99.5%), deuterium oxide (heavy water) and thiol and amine‐terminated polyethylene glycol (HS‐PEG5k‐NH_2_, *M*_n_ = 5000 g mol^−1^) were purchased from Sigma–Aldrich. Hydrogen tetrachloroaurate trihydrate (HAuCl_4_·3H_2_O, 99.99%) was purchased from abcr GmbH. Hexadecyltrimethylammonium bromide (CTAB, 99%) was purchased from Merck KGaA. Sylgard 184 polydimethylsiloxane (PDMS) elastomer kits were obtained from Dow Corning. AZ5214E photoresist and AZ351B developer were purchased from Microchemicals GmbH. Human TNF‐alpha (TNF‐α, premium grade, 130‐094‐014) and anti‐TNF‐alpha antibody (130‐095‐749) were obtained from Miltenyi Biotec. Fluorescent‐tagged anti‐human IgG secondary antibody (SA5‐10110) was obtained from Thermo Fisher. Ethanol (EtOH) was purchased from Carl Roth.

### 4.2 Methods

#### 4.2.1 Synthesis of CTAC AuNPs and Functionalization With Amine Groups

AuNPs were synthesized following a protocol as previously reported [38]. First, Wulff seeds were synthesized by tempering an aqueous solution of CTAB (4.7 mL, 100 mM) containing HAuCl_4_ (1.25 µmol) at 32°C for at least 10 min. Under vigorous stirring, freshly prepared NaBH_4_ (300 µL, 10 mM) was added in a swift one‐shot injection into the vortex. Stirring was discontinued after 30 s, and the seeds were stirred at 300 rpm for 30 min at 32°C. Afterward, CTAC (40 mL, 200 mM), ascorbic acid (30 mL, 100 mM), and the previously prepared Wulff‐seed solution (1 mL) were mixed. Then, 40 mL of a mixture of CTAC (200 mM) and HAuCl_4_ (0.5 mM) was added under modest stirring. After 15 min, the solution was centrifuged at 15000 rpm for 1 h, washed with water, centrifuged again at 15000 rpm for 1 h, and then redispersed in CTAC solution (10 mL, 20 mM). The resulting 8 nm seeds were then used in the next step in various amounts. A solution containing CTAC (200 mL, 60 mM) and ascorbic acid (1.3 mM) was mixed with the 8 nm seeds (840 µL).

Then, HAuCl_4_ solution (180 mL, 1 mM) was added continuously with a syringe pump system at a rate of 1.5 mL/min. Afterward, 20 mL of the same solution was added to the solution in a one‐shot injection. After 15 min, the solution was centrifuged at an appropriate speed (2000 rcf) for 20 min, washed with CTAC (10 mM), centrifuged again for 20 min, and then redispersed in CTAC (10 mM). Amine terminations were introduced to the synthesized particles using a modified protocol from established work [53]. HS‐PEG(5k)‐NH_2_ (2 mg) was dissolved in dichloromethane (DCM, 200 µl). 100 µL of this solution was added to 1 mL of the AuNPs (1 mg ml^−1^ in 1 mM CTAC). After thorough mixing, methanol (1 mL) was added, followed by further vigorous mixing and then venting. The mixture was shaken overnight to obtain the amine‐terminated gold nanospheres (AuNP‐NH_2_). Purification of the AuNP‐NH_2_ was performed by multiple cycles of centrifugation (2000 rpm, 30 min) and redispersion in a CTAC solution (200 µM).

#### 4.2.2 Characterization Techniques

AuNPs were characterized using transmission electron microscopy (TEM), dynamic light scattering (DLS), and zeta‐potential measurements. The quality of the fabricated PDMS templates was assessed using Atomic Force Microscopy (AFM), while that of the nanoparticle chains was investigated using Scanning Electron Microscopy (SEM). Transmission spectroscopy was conducted in the range of 400 to 1500 nm using a Cary 5000 spectrometer (Agilent, USA).

#### 4.2.3 Biofunctionalization of the AuNP Array

Wells of 5 mm diameter each, W1, W2, and W3, were filled with GA (2,5%) in EtOH (50%) and were incubated for 30 min. The wells were later washed 3 times with deionized (DI) water. W1 and W3 were further incubated with TNF‐α (40 µL, 50 µg ml^−1^) suspended in DI water. In contrast, W2 was incubated with 3 mg ml^−1^ BSA dissolved in 0.01 M phosphate‐buffered saline (PBS, pH 7.4). After removing the cytokine solution, the chip was washed with PBS (0.1 M, pH 7.4) supplemented with TWEEN 20 (0.05% v/v) to remove unbound proteins. In W1, a final blocking step was completed using BSA (3 mg ml^−1^) for 30 min to block the remaining binding sites. To confirm the desired surface modification, a fluorescent‐based immunodetection step was conducted. Firstly, we incubated W1 and W2 with anti‐human TNF‐α antibody (40 µL, 5 µg mL ^−1^) for 1 h. Next, the wells were washed 3 times with PBS (0.1 M, pH 7.4) supplemented with TWEEN 20 (0.05% v/v). To enable the detection of bound primary antibodies, the wells were then incubated for 1 h in the dark with fluorescent‐tagged anti‐human IgG secondary antibody (40 µL, 5 µg mL ^−1^). After washing the wells three times with PBS (0.1 M, pH 7.4), they were dried using a gentle air stream before being imaged for fluorescence.

#### 4.2.4 Finite Difference Time Domain Simulations

A commercial‐grade simulator based on the FDTD (Finite Difference Time Domain) method [61], was used to perform the calculations. This approach considered several assumptions. These assumptions and the geometry of the simulated system are described below. AuNPs were modeled by spherical structures with a diameter of 50 nm. To simulate the extinction spectrum of a single particle on a glass substrate, a total‐field scattered‐field source (with a wavelength range of 400–1200 nm) was employed, illuminating the sample at normal incidence. Symmetric and antisymmetric boundary conditions and perfectly matching layer boundary conditions were applied according to the polarization of the source. To check the sensitivity of the LSPR modes of the plasmonic particles to the refractive index of the surroundings, the background refractive index was varied from 1 to 1.5. The optical responses of such particles were obtained by using frequency‐domain field and power monitors. For the dielectric function of gold, data from Johnson and Christy [62] were fitted using six coefficients with a root mean‐squared (RMS) error of 0.22. The mesh size in the FDTD region was set to auto‐nonuniform, and an additional mesh overlay with a 1 nm mesh size was applied in all directions to enhance the simulation stability. Spherical particles were organized as dimer chains with a distance of 2 nm on a glass substrate. A plane‐wave source was used to simulate the optical response of plasmonic gratings, illuminating the structure at a normal incidence with a polarization angle of 0° representing the longitudinally polarized light (in line with the grating lines) and with a polarization angle of 90° representing the transversely polarized light (perpendicular to the grating lines). Broadband illumination (400–1200 nm) was collected through the transmission monitor. To calculate the extinction spectra of plasmonic gratings, the formula of “–ln(T)” was employed. Periodic boundary conditions were used along the X‐ and Y‐axis, and the periodicities were set according to experimental values along the X‐axis. Frequency domain field monitors were used to obtain the optical responses of the system. For the dielectric properties of glass, data from Palik [63], were utilized. For optimal simulation stability, the mesh area was set around the existing structure in all three principal directions with a mesh step size of 1 nm. The auto‐shutoff level was set to 10^−5^ for all simulations.

## Author Contributions

T.H.T. led data curation, visualization, and the writing of the original draft, and contributed equally to the investigation, formal analysis, and writing – review and editing. S. Sec. supported data curation, visualization, and original draft writing, and contributed equally to the investigation, formal analysis, and writing – review and editing. M.H. supported the investigation, formal analysis, original draft writing, and writing – review and editing. I.Ç. supported the investigation, original draft writing, and writing – review and editing. G.Y. supported the investigation and writing – review and editing. A.A. supported the formal analysis and writing – review and editing. S. Sar. supported the investigation, formal analysis, visualization, and writing – review and editing. C.R. supported supervision and writing – review and editing. T.A.F.K. supported supervision and writing – review and editing. A.F. supported conceptualization, funding acquisition, supervision, and writing – review and editing. L.B. led conceptualization, funding acquisition, and supervision, and supported the writing of the original draft and writing – review and editing. T.H.T. and S. Sec. contributed equally to this work, and all authors approved the final version of the manuscript.

## Funding

Larysa Baraban, Taufhik H. Tonmoy, and Andreas Fery acknowledge the financial support of DFG, German Research Foundation for the funding of project 451785257 (​GRK 2767 – ‘Supracolloidal Structures’) and project BA 4986/10. Larysa Baraban acknowledges the financial support of the project ImmunoChip, which has received funding from the European Research Council (ERC) under the European Union's Horizon Europe research and innovation program grant agreement No 101045415. Ahmed Alsadig thanks the Alexander von Humboldt Foundation for the support. The Volkswagen Foundation financially supported this project through a Freigeist Fellowship to Tobias A.F. König. Christian Rossner acknowledges receipt of a Liebig fellowship (Fonds der Chemischen Industrie).

## Conflicts of Interest

The authors declare no conflict of interest.

## Supporting information




**Supporting file 1**: smll71988‐sup‐0001‐SuppMat.pdf

## Data Availability

The data that support the findings of this study are available from the corresponding authors upon reasonable request.
