# Supplementary material for: Enhanced Refractive Index Sensitivity of Linearly Assembled Gold Nanoantennae for Biosensing Applications
Source: Small. 2025 Dec 23;22(9):e10159. doi: 10.1002/smll.202510159 (PMC12895226; doi:10.1002/smll.202510159)
Supplement: Supplementary file 1 — Supporting file 1: smll71988‐sup‐0001‐SuppMat.pdf [file SMLL-22-e10159-s001.pdf]

# Supporting Information

## Enhanced Refractive Index Sensitivity of Linearly Assembled Gold Nanoantennae for Biosensing Applications

*Taufhik H. Tonmoy**, *Sezer Seçkin, Marisa Hoffmann, Isli Çela, Gyusang Yi, Ahmed Alsadig, Swagato Sarkar, Christian Rossner, Tobias A.F. König, Andreas Fery, and Larysa Baraban**

Email: h.tonmoy@hzdr.de, l.baraban@hzdr.de

**Table S1**. Plasma Parameters Used to Produce PDMS Templates *via* Wrinkle Formation

| Sample ID | Periodicity (nm) | Depth (nm) | Exposure Time (s) | Plasma Pressure (mBar) |
|---|---|---|---|---|
| 101 | 390 | 62 | 240 | 0.7 |
| 103 | 400 | 70 | 210 | 0.7 |
| 105 | 370 | 65 | 210 | 0.9 |
| 107 | 440 | 80 | 300 | 0.7 |
| 109 | 460 | 85 | 360 | 0.7 |
| 111 | 339 | 48 | 180 | 0.7 |
| 113 | 330 | 45-55 | 150 | 0.7 |
| 115 | 297 | 40 | 120 | 0.7 |
| 118 | 279 | 36 | 120 | 0.9 |
| 120 | 252 | 31 | 60 | 0.9 |
| 122 | 265 | 30 | 60 | 0.7 |

S2. Preparation of PDMS Templates *via* Wrinkle Formation

Sylgard 184 base and curing agents were used in the ratio 5:1 to prepare PDMS slabs and cut into 1 cm x 4 cm pieces. A custom holder was used to stretch the PDMS uniformly. The stretched PDMS was placed in oxygen plasma (according to parameters in Table S1). Upon release of the PDMS from the holder, the oxygen plasma-exposed surface formed wrinkles due to a mismatch of Young's modulus between the hardened surface and bulk PDMS.

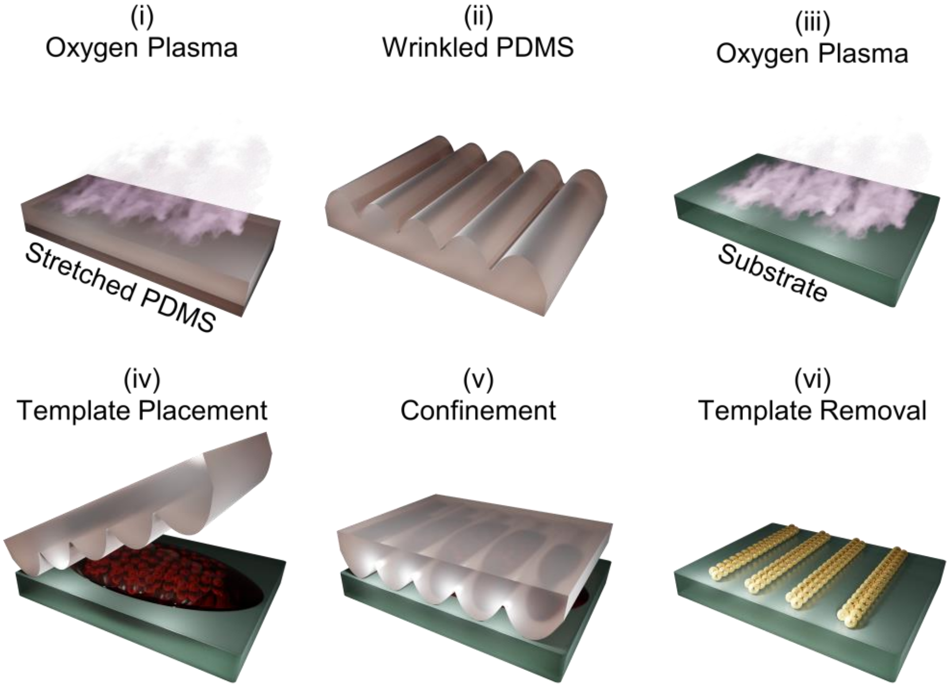


**Figure S2.** Fabrication of wrinkled PDMS template. (i) Pre-stretched PDMS is exposed to oxygen plasma which hardens the top surface (ii) Release of the stretched PDMS causes wrinkle formation due to Young's modulus mismatch between hardened top surface and bulk PDMS (iii) The target substrate is cleaned with acetone, ethanol and water, respectively, followed by blow-drying with nitrogen. Furthermore, its surface is hydrophilized using oxygen plasma (iv) Colloidal gold is placed on the substrate and covered with wrinkled PDMS template (iv) Drying of the colloidal gold under confinement causes capillary forces to assemble the particles into the cavities of the template (v) Removal of the template leaves the self-assembled particle chains on the substrate.

S3. Fabrication of PDMS Templates *via* Laser Interference Lithography (LIL)

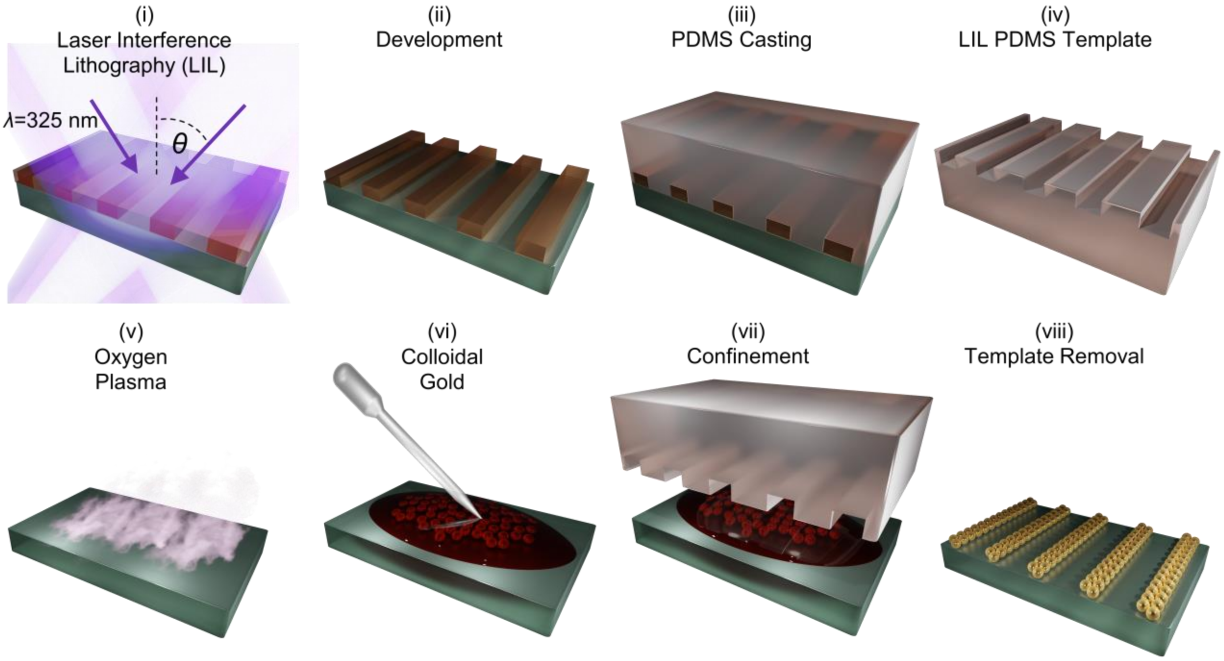


**Figure S3.** Laser interference lithography steps. (i) A laser beam is expanded, collimated, and split using a set of lenses and a beam splitter, respectively. The two beams are redirected using mirrors and made to interfere on a negative-tone photoresist on a substrate, exposing a periodic pattern because of the constructive and destructive interference between these two beams (ii) Development process selectively removes photoresist from unexposed areas (iii) PDMS is cast over the LIL master and cured (iv) The PDMS is removed from the master and is now ready as a template (v) The target substrate is cleaned with acetone, ethanol and water, respectively, followed by blow-drying with nitrogen. Furthermore, its surface is hydrophilized using oxygen plasma (vi) Colloidal gold is dropped on to the substrate and confined with PDMS template (vii) Drying of the colloidal gold under confinement results in capillary forces driving the gold nanoparticles into the cavities of the template (viii) Removal of the template leaves the self-assembled particle chains on the substrate.

S4 Finite difference time domain (FDTD) simulation results

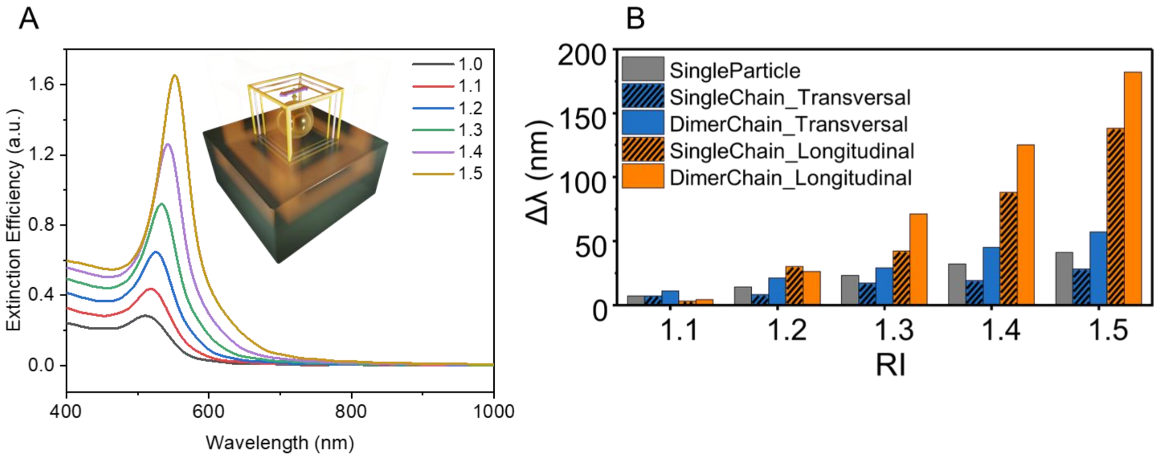


**Figure S4.** (A) Simulated spectra of isolated single gold-nanoparticle (50 nm) on glass substrate with varying refractive index. The spectra are polarization independent (B) Comparison of change in resonant peaks (Δλ) due to variation in refractive index (RI) for the case of isolated single particle, chains of single particles, and chains of dimers. The suffixes _Transversal and _Longitudinal indicate the polarization of light.

S5. Measurement Setup for Refractive Index Sensitivity

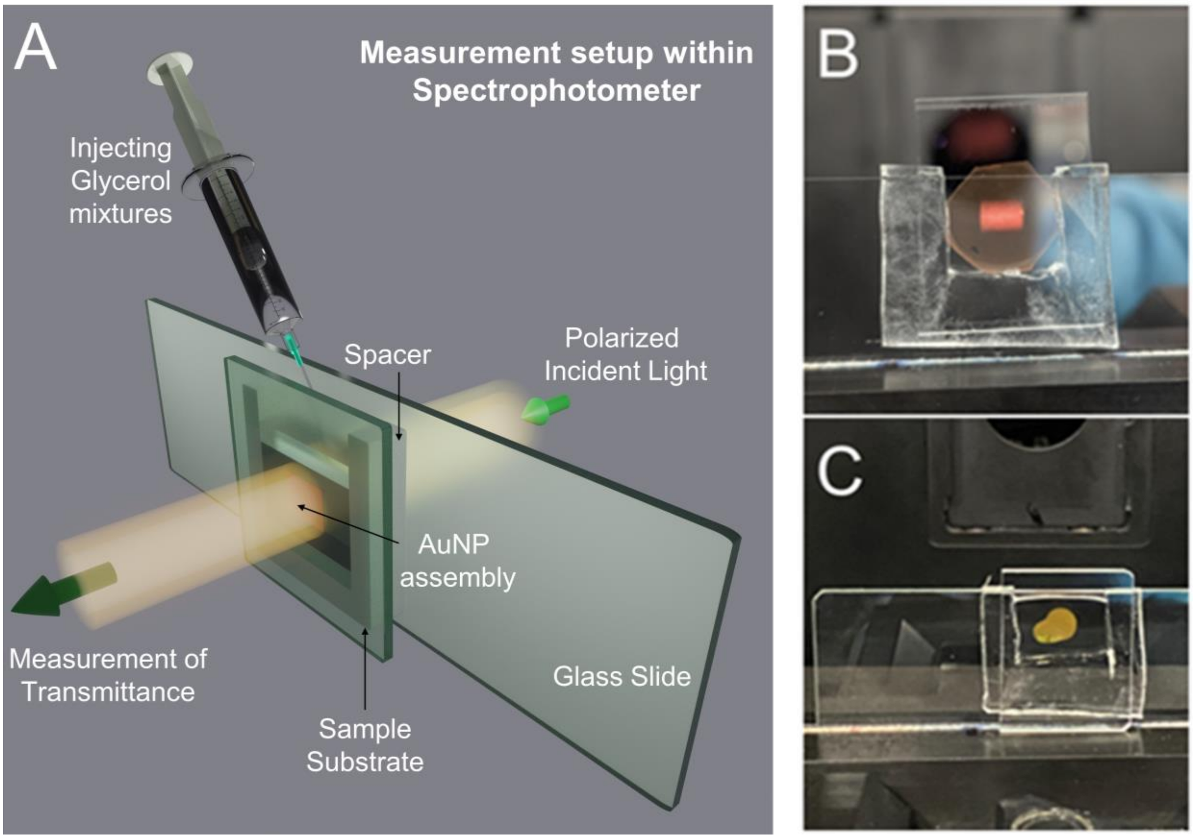


Figure S5. Measurement Setup. (A) Glass chip with AuNP assembly is attached to a reservoir made from a double-sided tape and another glass slide. The reservoir is kept empty to represent refractive index of 1.0 or filled with ~250 µl of deuterated water (heavy-water) containing different weights per volume (% w/v) of glycerol (B) Glass chip containing AuNP dimer-chains showing a diffraction pattern which changes color depending on the relative position of observer, chip and incident lights (C) drop-casted concentrated colloidal gold forming aggregates having a clear golden color.

Table S6. Refractive index collected using multi-wavelength digital refractometer

| | 0% | 1% | 2% | 5% | 7.5% | 10% | 25% | 50% | 75% |
|---|---|---|---|---|---|---|---|---|---|
| **404.7** | -- | -- | -- | -- | -- | -- | 1.37591 | 1.41616 | 1.44804 |
| **435.8** | -- | -- | -- | 1.34166 | 1.34538 | 1.34921 | 1.37314 | 1.41313 | 1.44475 |
| **486.1** | 1.33219 | 1.33343 | 1.33468 | 1.33866 | 1.34241 | 1.34631 | 1.37003 | 1.40963 | 1.44097 |
| **546.1** | 1.32975 | 1.331 | 1.33227 | 1.33622 | 1.33995 | 1.3438 | 1.36737 | 1.40667 | 1.43783 |
| **587.6** | 1.32849 | 1.32972 | 1.33099 | 1.3349 | 1.33863 | 1.34246 | 1.36597 | 1.40511 | 1.43615 |
| **589.3** | 1.32844 | 1.32967 | 1.33094 | 1.33485 | 1.33858 | 1.34241 | 1.36592 | 1.40506 | 1.43609 |
| **632.8** | 1.32733 | 1.32857 | 1.32983 | 1.33373 | 1.33745 | 1.34126 | 1.36471 | 1.40370 | 1.43462 |
| **656.3** | 1.32680 | 1.32805 | 1.32930 | 1.33321 | 1.33691 | 1.34073 | 1.36411 | 1.40303 | 1.43391 |
| **706.5** | 1.32581 | 1.32705 | 1.32830 | 1.33216 | 1.33586 | 1.33964 | 1.36298 | 1.40180 | 1.43264 |

A digital multi-wavelength refractometer was used to collect the refractive indices of different glycerol-deuterated water mixtures as a function of known wavelengths. Applying a Cauchy fit, the refractive index values were calculated separately, as presented in Figure S7 for each of these concentrations. The “A” values extracted from the Cauchy fits were used in the simulation as well as experiments to calculate the sensitivity of the different samples.

**S7.** Cauchy fits for digital refractometer data

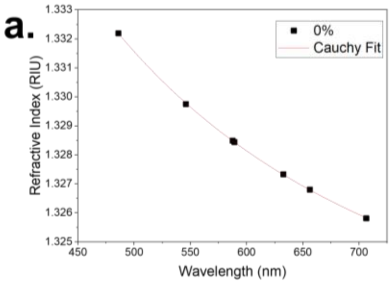


| | |
|---|---|
| Model | Cauchydispersion (User) |
| Equation | A+(B/(x^2))+ (C/(x^4)) |
| Plot | 0 percent |
| A | 1.31935 ± 1.62559E-4 |
| B | 3414.63062 ± 107.57701 |
| C | -9.04158E7 ± 1.70516E7 |
| Reduced Chi-Sqr | 5.00572E-10 |
| R-Square (COD) | 0.99992 |
| Adj. R-Square | 0.99989 |

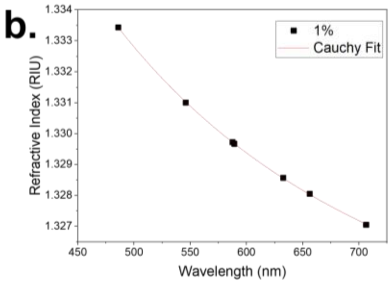


| | |
|---|---|
| Model | Cauchydispersion (User) |
| Equation | A+(B/(x^2))+ (C/(x^4)) |
| Plot | 1 percent |
| A | 1.32062 ± 1.45136E-4 |
| B | 3392.9681 ± 96.04709 |
| C | -8.70085E7 ± 1.5224E7 |
| Reduced Chi-Sqr | 3.99021E-10 |
| R-Square (COD) | 0.99994 |
| Adj. R-Square | 0.99991 |

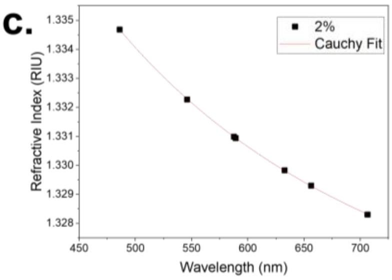


| | |
|---|---|
| Model | Cauchydispersion (User) |
| Equation | A+(B/(x^2))+ (C/(x^4)) |
| Plot | 2 percent |
| A | 1.32172 ± 1.21932E-4 |
| B | 3502.97381 ± 80.69164 |
| C | -1.04283E8 ± 1.27901E7 |
| Reduced Chi-Sqr | 2.81634E-10 |
| R-Square (COD) | 0.99996 |
| Adj. R-Square | 0.99994 |

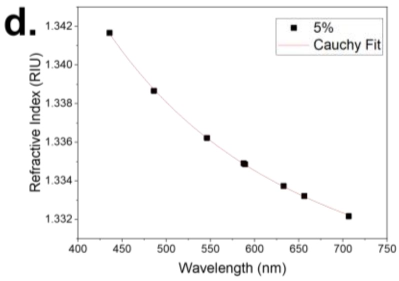


| | |
|---|---|
| Model | Cauchydispersion (User) |
| Equation | A+(B/(x^2))+ (C/(x^4)) |
| Plot | 5 percent |
| A | 1.32605 ± 2.42122E-4 |
| B | 3156.35045 ± 143.13942 |
| C | -3.75871E7 ± 1.94942E7 |
| Reduced Chi-Sqr | 2.78448E-9 |
| R-Square (COD) | 0.9998 |
| Adj. R-Square | 0.99972 |

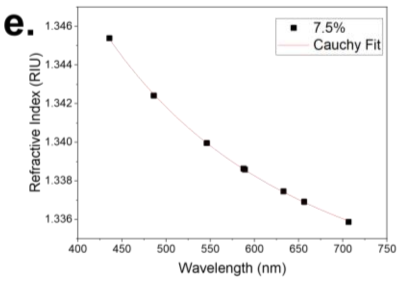


| | |
|---|---|
| Model | Cauchydispersion (User) |
| Equation | A+(B/(x^2))+ (C/(x^4)) |
| Plot | 7.5 percent |
| A | 1.3296 ± 2.23387E-4 |
| B | 3254.68164 ± 132.06328 |
| C | -4.99557E7 ± 1.79857E7 |
| Reduced Chi-Sqr | 2.37022E-9 |
| R-Square (COD) | 0.99983 |
| Adj. R-Square | 0.99976 |

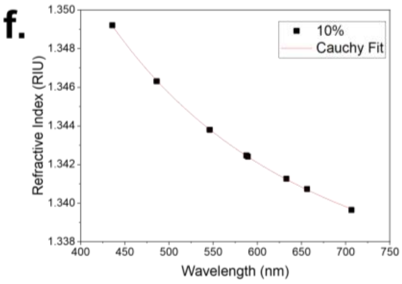


| | |
|---|---|
| Model | Cauchydispersion (User) |
| Equation | A+(B/(x^2))+ (C/(x^4)) |
| Plot | 10 percent |
| A | 1.3331 ± 1.82083E-4 |
| B | 3436.80046 ± 107.64524 |
| C | -7.23306E7 ± 1.46602E7 |
| Reduced Chi-Sqr | 1.57476E-9 |
| R-Square (COD) | 0.99989 |
| Adj. R-Square | 0.99984 |

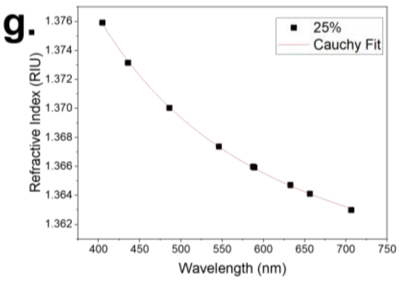


| | |
|---|---|
| Model | Cauchydispersion (User) |
| Equation | A+(B/(x^2))+ (C/(x^4)) |
| Plot | 25 percent |
| A | 1.35669 ± 3.11889E-4 |
| B | 3230.65726 ± 171.52859 |
| C | -1.56886E7 ± 2.10586E7 |
| Reduced Chi-Sqr | 8.03907E-9 |
| R-Square (COD) | 0.99968 |
| Adj. R-Square | 0.99958 |

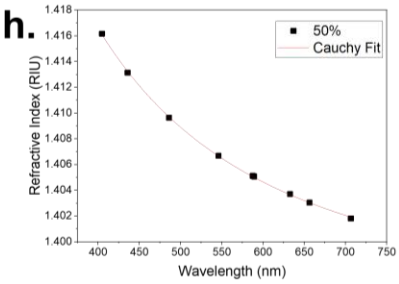


| | |
|---|---|
| Model | Cauchydispersion (User) |
| Equation | A+(B/(x^2))+ (C/(x^4)) |
| Plot | 50 percent |
| A | 1.39475 ± 2.96323E-4 |
| B | 3612.46435 ± 162.96783 |
| C | -1.93382E7 ± 2.00076E7 |
| Reduced Chi-Sqr | 7.25666E-9 |
| R-Square (COD) | 0.99977 |
| Adj. R-Square | 0.99969 |

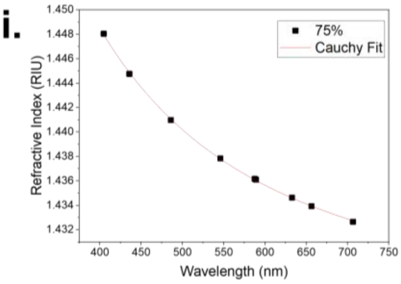


| | |
|---|---|
| Model | Cauchydispersion (User) |
| Equation | A+(B/(x^2))+ (C/(x^4)) |
| Plot | 75 percent |
| A | 1.42519 ± 3.18457E-4 |
| B | 3785.60135 ± 175.14074 |
| C | -9355563.375 ± 2.15021E7 |
| Reduced Chi-Sqr | 8.38122E-9 |
| R-Square (COD) | 0.99977 |
| Adj. R-Square | 0.99969 |

**Figure S7.** Cauchy fit for calculating refractive index from dispersion relation of varying glycerol concentrations

Table S8. Results for refractive index using "A" from Cauchy Fit data

| Glycerol Concentration | Cauchy Fit value (A) | 3-digit (for FDTD) |
|---|---|---|
| 0% | 1.31935 | 1.319 |
| 1% | 1.32062 | 1.321 |
| 2% | 1.32172 | 1.322 |
| 5% | 1.32605 | 1.326 |
| 7.5% | 1.32960 | 1.330 |
| 10% | 1.33310 | 1.333 |
| 25% | 1.35669 | 1.357 |
| 50% | 1.39475 | 1.394 |
| 75% | 1.42519 | 1.425 |

S9. Refractive Index Sensitivity and reproducibility measurements across various samples

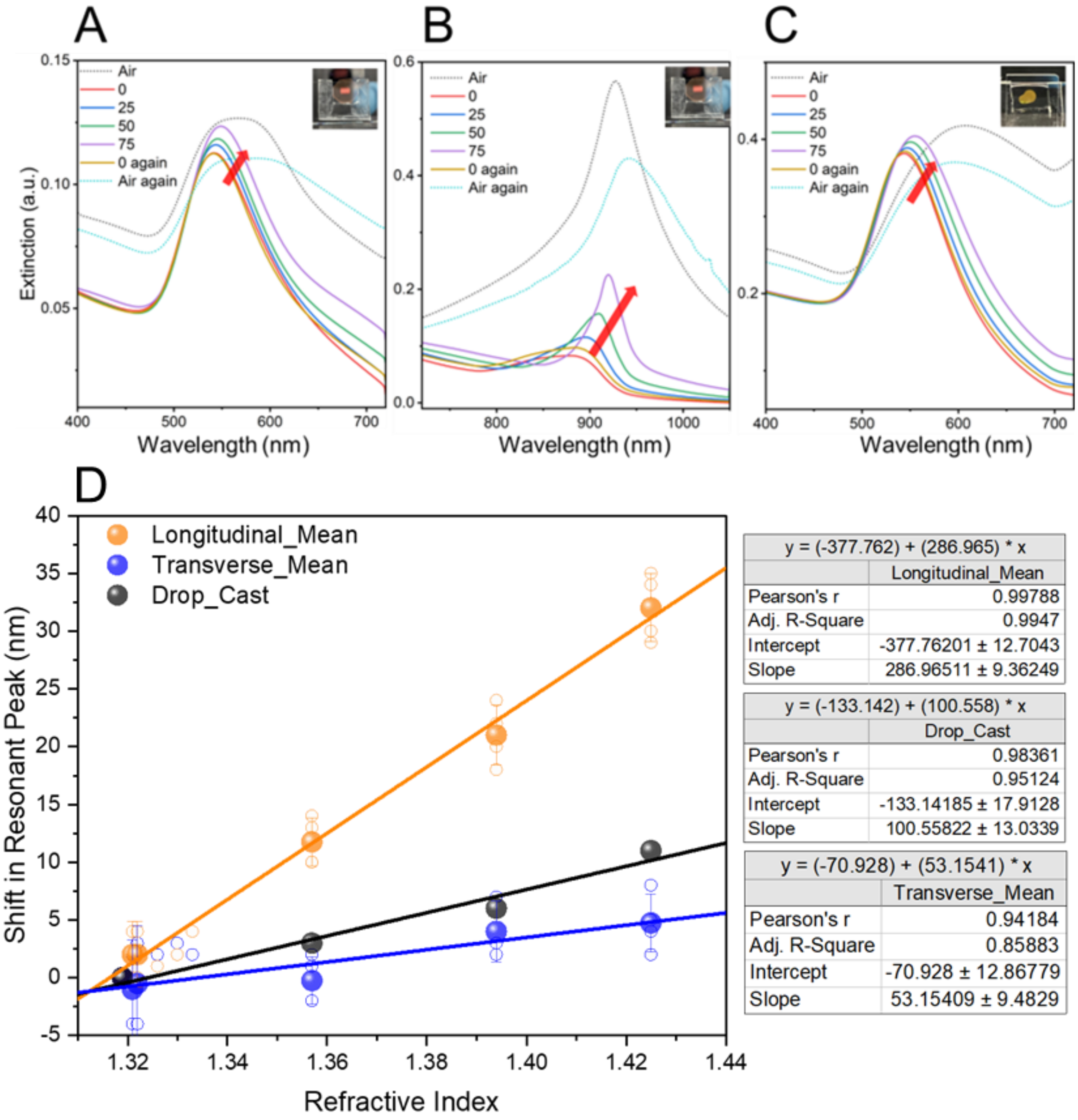


**Figure S9.** Refractive Index Sensitivity Measurements across various samples. (A) Dimer-chains, transversal polarized light (B) Dimer-chains, longitudinally polarized light (C) drop-casted gold nanoparticles with polarization independent spectra (D) Shift in resonant peak vs refractive index for different samples

S10. Effect of inter-particle distance, contributing to Blue Shift

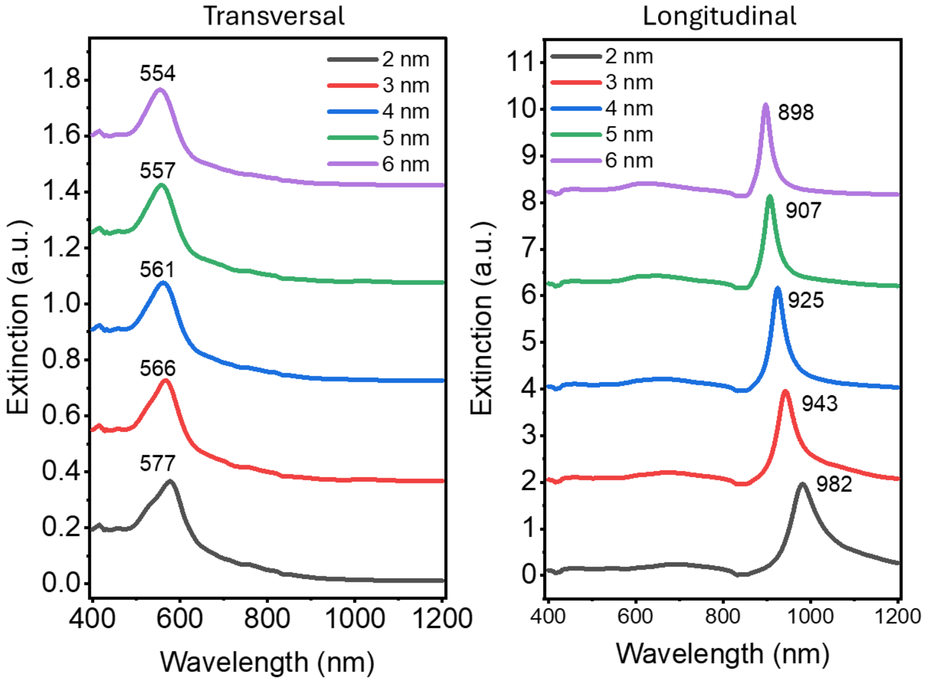


**Figure S10.** "Blue-shift" phenomenon: finite-difference time-domain (FDTD) simulation results for the effect of increased interparticle distance in a dimer chain of 10 units. The distance is increased in both x and y directions.

S11. Multiple cycles of refractive index change on the same sample as reproducibility and stability evidence.

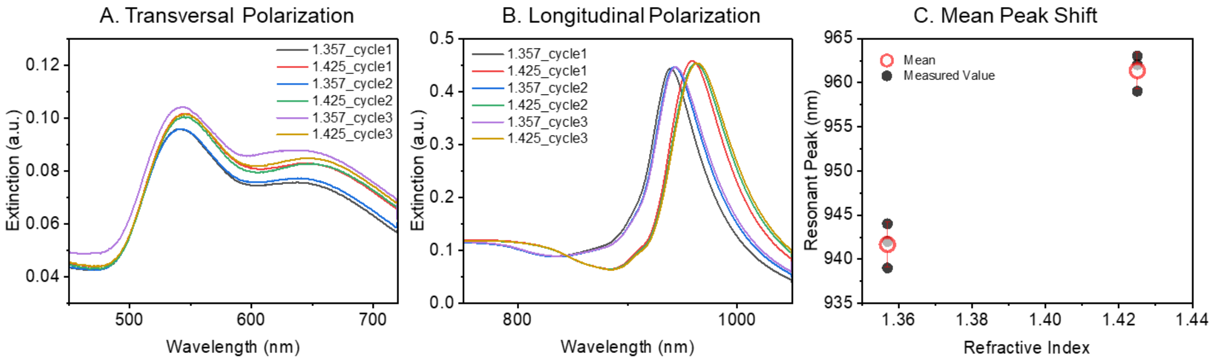


**Figure S11.** Polarized light vis-NIR spectra when the refractive index is cycled between 1.357 and 1.425 multiple times using corresponding concentrations of glycerol in deuterated water. (A) Spectra in case of transversal polarization of light; (B) spectra in case of longitudinal polarization of light; (C) Resonant peak wavelength vs refractive index graph showing clearly distinguishable levels in case of longitudinal polarization.

S12. Fluorescence Microscopy: Image Processing

Images were captured using a fluorescence microscope (Zeiss) using a standard green filter (488 nm) with 40x magnification lens and 4000 ms exposure. The images were converted to 8 bit using ImageJ software and the threshold was adjusted such that a window before any functionalization is carried out (Figure S6A) has a mean gray value of 0 for a square area of 2002 $\mu m^2$ near the center of the image (that is- photoresist window). The same threshold was then applied to all other images (Figure S6B illustrating one measurement with complete functionalization) and the minimum, maximum and mean gray-value were extracted for each case. For illustration, a green overlay was added to the images.

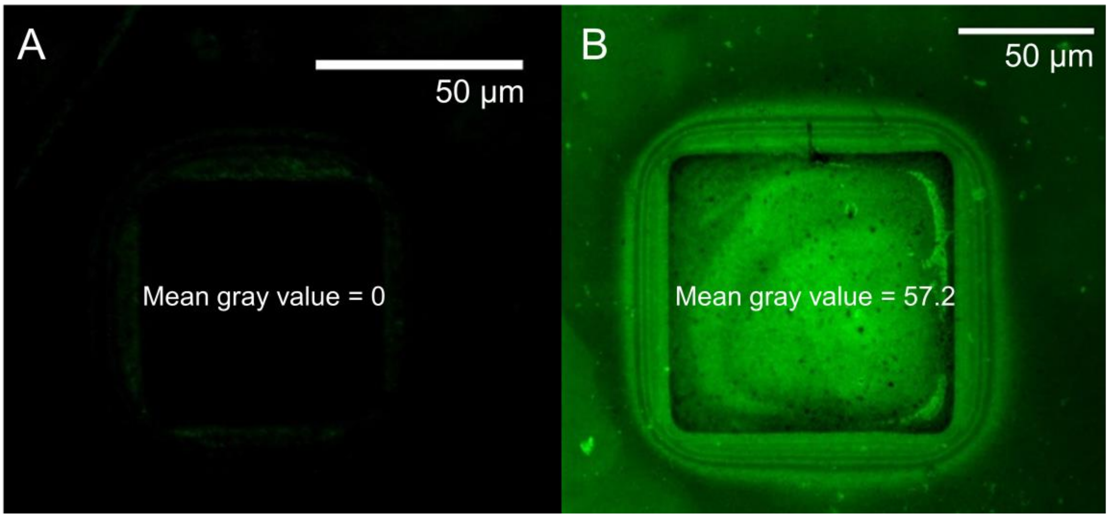


**Figure S12.** Images processed with ImageJ software showing (A) threshold adjusted such that the mean gray value near center of photoresist window for the case of gold nanoparticle lines is 0 and (B) same threshold applied to the case of lines after biofunctionalization and primary and secondary antibody attachment shows higher mean gray value.

S13. Polarized light vis-NIR spectra showing the effect of exposure to serum on bovine serum albumin (BSA)-passivated surface

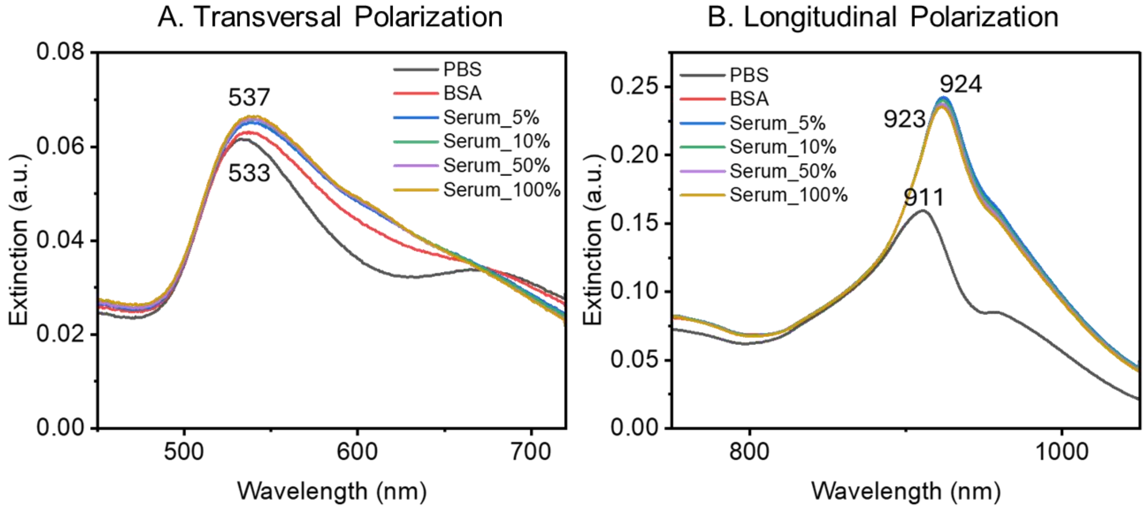


**Figure S13.** Polarized light vis-NIR spectra showing the effect of exposure to serum on bovine serum albumin (BSA)-passivated surface for transversal (A) and longitudinal (B) polarization of light.

Each serum concentration was incubated during 30 minutes. The chip was then washed gently with PBS three times. Finally, the vis-NIR spectra was recorded in PBS to maintain a fixed background medium. Initially, the BSA step exhibited a clearly measurable resonant peak shift (12 nm for longitudinal case) which corresponds to its adsorption on the sensor surface. After this passivation step, successive exposure to increasing serum concentrations did not show any measurable impact. From these results, we deduce that the concentration and incubation time for BSA was sufficient for complete passivation of the surface and protected it from further influence from the serum samples.

S14. Calibration curve of the ELISA assay for the TNF-α and capture antibody

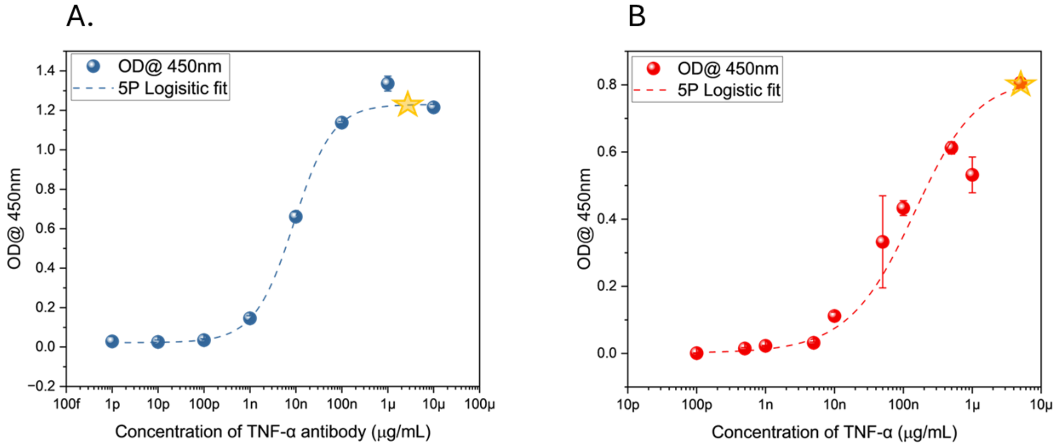


**Figure S14.** Calibration curves of the ELISA assay for the TNF-α and capture antibody (A) and TNF-α (B) measured at 450 nm. The yellow star indicates the antibody concentration (5 µg/mL). We have used 50 µg/mL in our plasmonic sensor experiments, which lies at the upper plateau of the ELISA response, confirming that the concentration employed in the proof-of-concept study corresponds to a region well above the detection limit of the commercial ELISA assay.

S15. Effect of particle removal in a dimer chain of 10 units (simulation results)

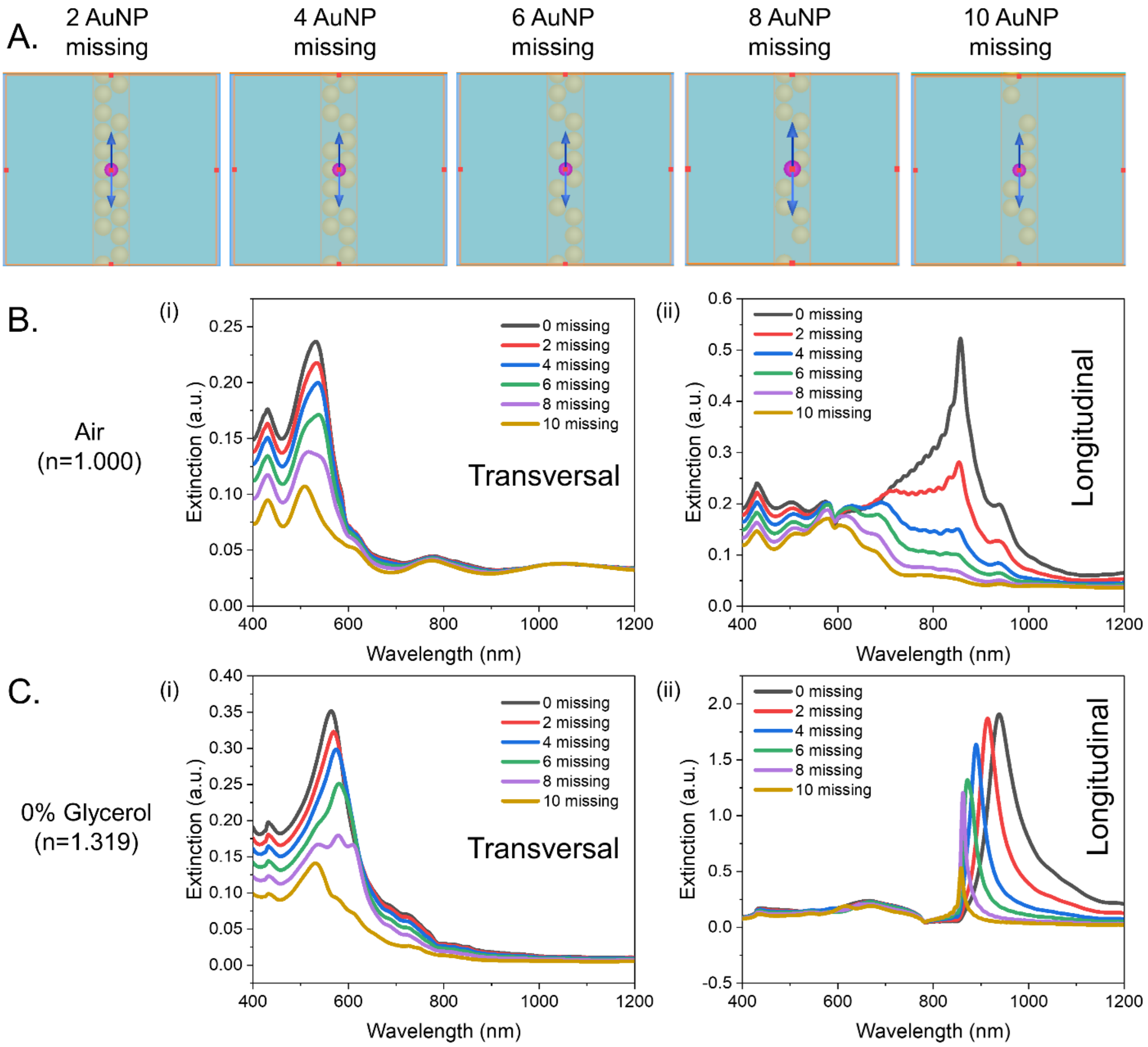


**Figure S15.** FDTD simulation snapshots for different cases of particle removal, varying from 2 to 10 Au nanoparticles in increments of two, selected randomly from the unit. Simulations were performed for both air and 0% glycerol environments, as well as under transverse and longitudinal polarizations. As expected, particle removal caused negligible shifts in the transverse plasmon modes but resulted in a reduction in overall extinction intensity, primarily due to the decreased effective cross-sectional area. In contrast, a distinct blue shift was observed, particularly in the 0% glycerol environment, indicating the emergence of plasmonic oligomer modes resulting from particle removal. For the air case, the extinction peak corresponding to these modes diminished and eventually vanished, confirming that the oligomeric plasmon modes are highly sensitive to the refractive index of the surrounding medium and persist only in higher-index environments.
